# Association of *APOE* ε4 genotype and lifestyle with cognitive function among Chinese adults aged 80 years and older: A cross-sectional study

**DOI:** 10.1371/journal.pmed.1003597

**Published:** 2021-06-01

**Authors:** Xurui Jin, Wanying He, Yan Zhang, Enying Gong, Zhangming Niu, John Ji, Yaxi Li, Yi Zeng, Lijing L. Yan

**Affiliations:** 1 Global Health Research Center, Duke Kunshan University, Kunshan, Jiangsu, China; 2 MindRank AI Ltd., Hangzhou, China; 3 School of Population and Global Health, University of Melbourne, Melbourne, Australia; 4 Environmental Research Center, Duke Kunshan University, Kunshan, China; 5 Duke Global Health Institute, Duke University, Durham, North Carolina, United States of America; 6 Center for Healthy Aging and Development Studies, National School of Development, Peking University, Beijing, China; 7 Center for the Study of Aging and Human Development and Geriatrics Division, Medical School of Duke University, Durham, North Carolina, United States of America; 8 Department of Preventive Medicine, Feinberg School of Medicine, Northwestern University, Chicago, Illinois, United States of America; 9 The George Institute for Global Health, Beijing, China; 10 School of Health Sciences, Wuhan University, Wuhan, China; University of Cambridge, UNITED KINGDOM

## Abstract

**Background:**

Apolipoprotein E (*APOE*) ε4 is the single most important genetic risk factor for cognitive impairment and Alzheimer disease (AD), while lifestyle factors such as smoking, drinking, diet, and physical activity also have impact on cognition. The goal of the study is to investigate whether the association between lifestyle and cognition varies by *APOE* genotype among the oldest old.

**Methods and findings:**

We used the cross-sectional data including 6,160 oldest old (aged 80 years old or older) from the genetic substudy of the Chinese Longitudinal Healthy Longevity Survey (CLHLS) which is a national wide cohort study that began in 1998 with follow-up surveys every 2–3 years. Cognitive impairment was defined as a Mini-Mental State Examination (MMSE) score less than 18. Healthy lifestyle profile was classified into 3 groups by a composite measure including smoking, alcohol consumption, dietary pattern, physical activity, and body weight. *APOE* genotype was categorized as *APOE* ε4 carriers versus noncarriers. We examined the associations of cognitive impairment with lifestyle profile and *APOE* genotype using multivariable logistic regressions, controlling for age, sex, education, marital status, residence, disability, and numbers of chronic conditions.

The mean age of our study sample was 90.1 (standard deviation [SD], 7.2) years (range 80–113); 57.6% were women, and 17.5% were *APOE* ε4 carriers. The mean MMSE score was 21.4 (SD: 9.2), and 25.0% had cognitive impairment. Compared with those with an unhealthy lifestyle, participants with intermediate and healthy lifestyle profiles were associated with 28% (95% confidence interval [CI]: 16%–38%, *P* < 0.001) and 55% (95% CI: 44%–64%, *P* < 0.001) lower adjusted odds of cognitive impairment. Carrying the *APOE* ε4 allele was associated with 17% higher odds (95% CI: 1%–31%, *P* = 0.042) of being cognitively impaired in the adjusted model. The association between lifestyle profiles and cognitive function did not vary significantly by *APOE* ε4 genotype (noncarriers: 0.47 [0.37–0.60] healthy versus unhealthy; carriers: 0.33 [0.18–0.58], *P* for interaction = 0.30). The main limitation was the lifestyle measurements were self-reported and were nonspecific. Generalizability of the findings is another limitation because the study sample was from the oldest old in China, with unique characteristics such as low body weight compared to populations in high-income countries.

**Conclusions:**

In this study, we observed that healthier lifestyle was associated with better cognitive function among the oldest old regardless of *APOE* genotype. Our findings may inform the cognitive outlook for those oldest old with high genetic risk of cognitive impairment.

## Abstract 2

In a cross-sectional study, Xurui Jin and colleagues study associations between cognition, lifestyle factors, and APOE genotype among adults aged 80 years or older in China.

## Author summary

**Why was this study done?:**

- Both genetic and lifestyle factors have impact on cognitive function among older adults, but previous studies showed inconsistent results on the interaction between lifestyle profiles and genetic risk on cognition.
- One recent study found that healthy lifestyle profiles were associated with lower risks of dementia only among apolipoprotein E (*APOE*) ε4 noncarriers, but not among carriers. However, relationships between *APOE* ε4 carrier status, lifestyle factors, and cognitive function are not well understood for the oldest group of older adults.

**What did the researchers do and find?:**

- We used data from 6,160 participants 80 years or older from the Chinese Longitudinal Healthy Longevity Survey (CLHLS) with information on *APOE* genotype, lifestyles, and cognition.
- We defined lifestyle profile by a healthy lifestyle score including smoking, alcohol consumption, body weight, dietary pattern, and physical activity.
- Both healthy lifestyle profile and lower genetic dementia risk, represented by not carrying the *APOE* ε4 allele, were associated with lower odds of cognitive impairment.
- No interaction between *APOE* ε4 genotype and lifestyle profiles was observed.

**What do these findings mean?:**

- Our results suggest the importance of a healthier lifestyle for cognition regardless of genetic dementia risk and increases our understanding of this relationship in the oldest older adults (80 years and older).
- The main limitation of our study is that our data are cross-sectional and thus validation by cohort studies or trials are needed.

## Introduction

Genetic profile and lifestyle factors are both associated with cognitive function [1,2]. Among various genetic factors, the apolipoprotein E (*APOE*) ε4 allele is the most significant one, accounting for about 5% of the variance in lifetime cognitive decline and 4% of the variance in Alzheimer disease (AD) [3]. *APOE* is associated with the clearance rate of amyloid beta, which is a hallmark of AD, and the presence of the ε4 allele indicates a slower clearance rate [4].

The influence of modifiable lifestyle factors on cognition has been demonstrated in many studies [5–7]. Prior studies have proved that a healthier lifestyle characterized by abstaining from smoking or drinking, adhering to regular physical activity, and a healthy dietary pattern was associated with a low incidence of cognitive impairment [5,8–10]. However, most previous research examined single lifestyle factors; only a few studies investigated the impact of combined lifestyle profile on cognition. For instance, a study that included 977 Korean adults aged over 65 years old demonstrated that a healthier lifestyle profile was associated with a lower rate of cognitive decline [11]. In addition, an incremental benefit of multiple lifestyle behaviors was observed. But it is still unclear how a healthy lifestyle may contribute to the cognitive function among those oldest old (aged 80 years old or older), who represent the fastest-growing segment of populations worldwide.

Whether the effect of lifestyle on cognition varies by genetic dementia risk represented by different *APOE* genotypes is still unclear. Prior research reported inconsistent results on the interactions between *APOE* genotype and lifestyle factors on cognitive outcomes. Some studies found significant interactions between *APOE* genotype and single lifestyle factors, such as physical activity [12], alcohol consumption [13], and diet [14], or the combined effect of lifestyle factors [8], with stronger effects among *APOE* ε4 carriers than noncarriers. However, other studies failed to detect such an interaction [9]. All of these studies were conducted among middle-aged adults or older adults mostly under 80 years old.

We used the genetic substudy from the Chinese Longitudinal Healthy Longevity Survey (CLHLS), a large-scale community-based study, to examine whether *APOE* genotype interacts with the relationship between a combined lifestyle profile, including smoking, alcohol consumption, physical activity, dietary pattern, and body weight, with cognitive function among the oldest old in China. Together with other studies and evidence from interventional trials, the goal of our study is to shed light on potential health intervention strategies to improve cognitive functioning in older age, including the oldest old—the fastest-growing segment of our population.

## Methods

### Study design and sample

The present study uses data from the CLHLS, which is an ongoing longitudinal study that began in 1998 with follow-up surveys every 2 to 3 years. The CLHLS is a Chinese nationwide survey conducted in randomly selected counties and cities in 22 out of 30 provinces covering 85% of China’s population. All centenarians from the selected areas who agreed to participate were included in the study. Based on sex and place of residence (i.e., living in the same street, village, city, or county) for a given centenarian, randomly selected octogenarians and nonagenarians were also sampled. More details about the sampling procedure and quality of data of this survey have been published elsewhere [15]. Ethics approval was obtained from the Research Ethics Committees of Peking University and Duke University (IRB00001052-13074). All participants or their legal representatives signed written consent forms in the baseline and follow-up surveys.

In this cross-sectional analysis, we pooled baseline data from all CLHLS surveys conducted between 1998 and 2014 that included genetic and lifestyle assessment, and this study is reported as per the Strengthening the Reporting of Observational Studies in Epidemiology (STROBE) guidelines (S1 Checklist). The CLHLS genetic substudy had a total of 6,399 participants aged over 80 years or older. After excluding those participants with missing value for *APOE* genotyping (rs429358 or rs7412) (*N* = 133) or missing value in Mini-Mental State Examination (MMSE) or lifestyle measurements (*N* = 106; MMSE: 51, lifestyle factors: 55), we included 6,160 participants aged 80 or above in our analyses.

### *APOE* genotyping

Saliva samples were collected from participants in 6 waves of CLHLS (1998, 2000, 2002, 2005, 2008, and 2011), and the genotyping was performed in 2014 by Beijing Genomics Institute (BGI). A customized chip targeting about 27K longevity phenotype–related SNPs was used, and the BGI genotyping quality control procedure of the CLHLS genetics study is published elsewhere [16]. We extracted rs429358 and rs7412 to determine *APOE* genotype and further grouped all study participants by carrying the *APOE* ε4 allele (ε2/ε4, ε3/ε4, ε4/ε4 genotypes) or not (ε2/ε2, ε2/ε3, ε3/ε3 genotypes) [17].

### Assessment of healthy lifestyle

The data were collected through face-to-face interviews by trained interviewers who are local staff members from the county-level network system of the National Bureau of Statistics of China. All interviewers have received 12+ years of schooling, and most have earned a college degree. Each interviewer was accompanied by a local doctor, a nurse, or a medical college student so that some health checkups could be performed. In the physical examination, body weight and height were measured by trained medical staff using a standardized protocol. Survey questions from CLHLS were presented in S3 Text.

The healthy lifestyle score was constructed by collecting information on smoking, alcohol consumption, physical activity, dietary pattern, and body weight. The smoking status was categorized as current, former, or never smokers. Alcohol consumption status was categorized as binge, moderate, or never drinkers. Binge drinker status was defined as a current drinker with alcohol consumption of greater than 25 grams of alcohol per day for men and 15 grams per day for women. Moderate drinker status was defined as a current drinker with alcohol consumption of less than or equal to 25 grams per day for men and 15 grams per day for women. Never drinker status was those who self-reported never having had regular alcohol consumption. Physical activity was defined by 2 questions—“exercise or not at present?” and “exercised or not in the past?.” If the participants answered “Yes” to “exercise or not at present?” regardless of past exercise status, the physical activity was defined as “current.” If the answers were “No” for both questions, the physical activity was defined as “never.” If the participants answered “No” for “exercise or not at present?” and “Yes” for “exercised or not in the past?,” the physical activity status was defined as “former.” Dietary pattern was categorized as unfavorable, intermediate, or favorable by a simplified healthy eating index based on intake frequency of 5 food categories including fruits, vegetables, fish, bean products, and tea, which were demonstrated to be associated with cognitive function [18,19]. A 3-point scale question was used to measure the current intake frequency of each food group: “always or almost every day,” “sometimes or occasionally,” or “rarely or never.” Those 3 terms received a score of 2, 1, or 0, respectively, with higher scores indicating higher consumption. The scores of the intake of the 5 foods were summed and categorized into 3 categories: unfavorable: 0 to 4; intermediate: 5 to 6; and favorable: 7 to 10. Totally, 34.6% of the participants have missing value in body mass index (BMI) because height was not measured before the 2005 CLHLS survey. We used body weight instead of BMI to build the healthy lifestyle score and included BMI in the sensitivity analysis. Logistic regression model with penalized splines evaluated nonlinear associations of body weight with cognitive impairment [20]; 2 weight cutoffs were identified (weight less than 38 kg or higher than 50 kg) above and below, in which there was no significant increase in the magnitude of odds ratio for cognitive impairment (**S1A Fig**).

We assigned 0, 1, or 2 points for each group of the five 3-group lifestyle factors with higher score indicating a healthier lifestyle. The healthy lifestyle score was the sum of the scores of these 5 health-related factors ranging from 0 to 10. Three healthy lifestyle score cutoffs were identified (healthy: 8 to 10, intermediate: 6 to 7, and unhealthy: <6); among each cutoff, in which there was no significant change in the multitude of hazard ratio (HR) for mortality (**S2A Fig**).

### Assessment of cognitive function

**Fig 1:**
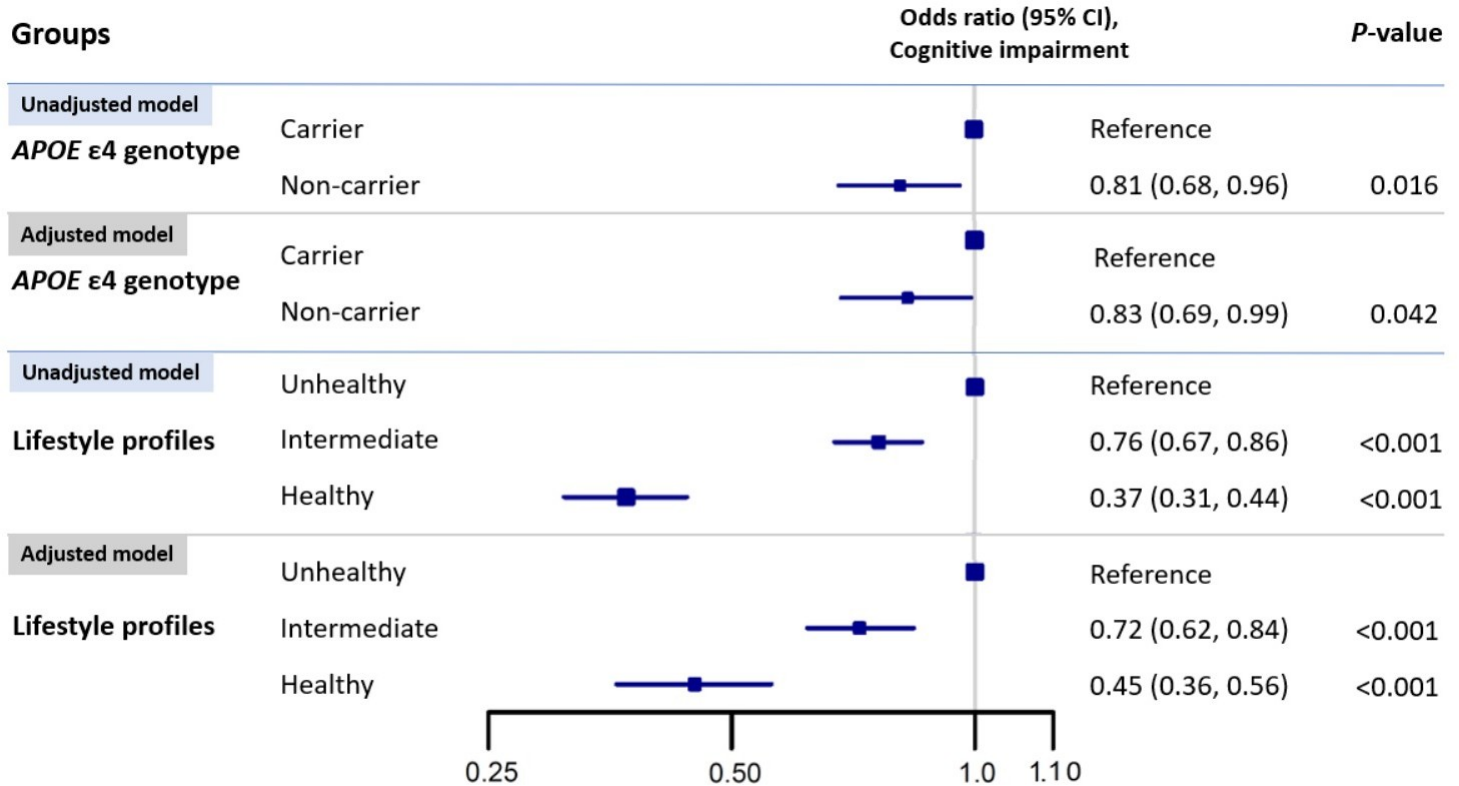
Associations* of cognitive impairment** with *APOE* ε4 genotype and lifestyle profiles. *Model adjusted for age, sex, residence, education level, marital status, *APOE* genotype, lifestyle profile, activity of daily living, and 7 kinds of self-reported disease (COPD, tuberculosis, cancer, diabetes, hypertension, stroke, and cardiovascular disease). **Cognitive impairment: cognitive impairment was defined by an MMSE score of less than 18. *APOE*, apolipoprotein E; CI, confidence interval; COPD, chronic obstructive pulmonary disease; MMSE, Mini-Mental State Examination.

The cognitive function of CLHLS participants was assessed by the Chinese version of the MMSE through a home-based interview, which includes 24 items, covering 7 subscales including orientation (4 points for time orientation and 1 point for place orientation); naming foods (naming as many kinds of food as possible in 1 minute, 7 points); registration of 3 words (3 points); attention and calculation (mentally subtracting 3 iteratively from 20, 5 points); copy a figure (1 point); recall (delayed recall of the 3 words mentioned above, 3 points); and language (2 points for naming objectives, 1 point for repeating a sentence, and 3 points for listening and following directions). The MMSE score ranges from 0 to 30. Higher scores represent a better cognitive function. The validity and reliability of this Chinese MMSE has been verified in several previous studies [21,22]. Consistent with previous studies [23], because a high proportion of our participants did not have formal education (approximately 70%), cognitive impairment was defined as an MMSE score of less than 18.

### Individual-level covariates

During each interview, the assessors measured a range of demographic, behavioral, and socioeconomic covariates. Following the previous studies, we consider potential confounders, including age, sex, ethnicity (Han versus others), residence (rural versus urban), main occupation before age 60 (nonmanual versus manual), marital status (currently married and living with spouse versus widowed, separated, divorced, or never married), education background (years of schooling: none versus ≥1 years of schooling), activity of daily living (need any assistance in bathing/dressing/toileting/transferring/eating/continence: impaired versus not impaired), and 7 kinds of self-reported diseases (chronic obstructive pulmonary disease [COPD], tuberculosis, all-cause cancer, diabetes, hypertension, stroke, and cardiovascular disease). All self-reported information was collected through face-to-face home interview by trained research staff members. Interviewees were encouraged to answer as many questions as possible. If they were unable to answer questions, a close family member or another proxy, such as a primary caregiver, provided answers [24].

### Statistical analysis

The development of the statistical analysis plan is described in S2 Text. Selected characteristics were presented as mean and standard deviation (SD) (continuous variables) or frequency distribution (categorical variable) by *APOE* phenotype (*APOE* ε4 carriers versus noncarriers). ANOVA for continuous variables and chi-squared tests for categorical variables were applied to test the significance levels of the differences. The association between healthy lifestyle, *APOE* phenotype, and cognitive function was assessed using multivariable logistic regression. The model adjusted for age, sex, residency, education level and marital status, activity of daily living, and 7 kinds of self-reported diseases.

In subgroup analyses, we examined whether the associations of cognitive impairment with lifestyle profiles differed by *APOE* ε4 genotypes in the multivariable-adjusted logistic regression model. The significance of multiplicative interactions was tested by cross product terms in the models. A 2-tailed *P* value of less than 0.05 was considered statistically significant.

We conducted 9 sensitivity analyses to check the robustness of the results, and the methodology details were included in S1 Text. The sensitivity analyses were carried out (1) using the longitudinal cognitive decline as the outcome to reduce the bias related to the cross-sectional design; and (2) using different MMSE cutoff scores (lower than 16, 21, or 25) to define cognitive impairment. The cutoff of 25 was normally the definition of cognitive impairment used in the younger population [23]. The cutoff of 16 and 21 was near our definition (lower than 18). Additionally, we adopted cutoff scores based on education level, which is widely accepted and used in China to define cognitive impairment (<18 for those without formal education, <21 for those with 1 to 6 years of education, and <25 for those with more than 6 years of education) to make the outcome (cognitive impairment) more interpretable [25,26]; (3) incorporating blood pressure and diabetes into the lifestyle score to build a modifiable factor score to make the score more replicable with other well-established lifestyle scores such as Cardiovascular Health Metric [27] or Pooled Cohort Equations [28] and using this score to reproduce the analysis; and (4) mapping the MMSE score to Clinical Dementia Rating (CDR) (MMSE score: 30 [CDR stage: 0], MMSE score: 26 to 29 [CDR stage: 0.5], MMSE score: 21 to 25 [CDR stage: 1], MMSE score: 11 to 20 [CDR stage: 2], and MMSE score 0 to 10 [CDR stage: 3]) [29] and using the CDR as outcome and ordinal logistics model to reproduce the analysis for increasing the statistical power. We also applied linear regression with MMSE score as outcome to reproduce our analysis, (5) excluding those who died within 2 years of the baseline surveys; (6) excluding the participants with an MMSE score equal to 0; (7) excluding the participants with deafness or blindness for those participants may have a low MMSE score because of the deafness or blindness; (8) using BMI to build the healthy lifestyle score instead of body weight, and the cutoff points were identified by a logistics regression model with penalized splines (0 point: <18 kg/m^2^, 1 point: 18 to 21 kg/m^2^, 2 points: >21 kg/m^2^) (**S1B Fig**); and (9) repeating the analyses using Poisson regression models to further add the statistical power.

The analyses were performed using STATA version 14.0 (Stata, College Station, Texas, United States of America) and R (version 3.61).

## Results

### Baseline characteristics of participants

Characteristics of the study sample are presented in **Table 1**. A total of 6,160 participants were included after excluding 239 participants that lacked genetic (*N* = 133), cognitive (*N* = 51), or lifestyle (*N* = 59) assessment. The total study sample comprised 1,076 *APOE* ε4 carriers (17.5%) and 5,084 *APOE* ε4 noncarriers (82.5%) with an average age of 90.1 ± 7.2 years, and among them, 1,267 (24.9%) had cognitive impairment. Participants with *APOE* ε4 genotype were more likely to be younger (mean age: 89.8 versus 90.8) and men (men: 45.4% versus 41.7%) (**Table 1**).

**Table 1:** Baseline characteristics of the 6,160 participants by *APOE* ɛ4 genotype.

Characteristics^a^	Total sample *N* = 6,160	*APOE* ɛ4 genotype		
		Carrier *N* = 1,076	Noncarrier *N* = 5,084	*P* value^b^
**Age, mean ± SD**	90.1 ± 7.2	89.8 ± 7.0	90.8 ± 7.3	<0.001
**Sex**				0.028
Men	2,609 (42.3)	488 (45.4)	2,121 (41.7)	
Women	3,551 (57.6)	588 (54.6)	2,963 (58.3)	
**Residence**				0.91
Urban	2,126 (34.5)	373 (34.7)	1,753 (34.5)	
Rural	4,034 (65.5)	703 (65.3)	3,331 (65.5)	
**Main occupation before age 60**^**c**^				0.68
Nonmanual	378 (6.8)	69 (6.4)	309 (6.1)	
Manual	5,782 (93.9)	1,007 (93.6)	4,775 (93.9)	
**Education background (school years)**				0.27
None (0)	4,271 (69.3)	731 (67.9)	3,540 (69.7)	
Ever (≥1)	1,889 (30.7)	345 (32.1)	1,488 (29.3)	
**Marital status**				0.38
Married (spouse alive)	1,380 (22.4)	252 (23.4)	1,128 (22.2)	
Others	4,778 (77.6)	824 (76.6)	3,954 (77.8)	
**Ethnicity**				0.74
Han	5,739 (93.1)	1,000 (92.9)	4,739 (93.2)	
Others (minority)	421 (6.8)	76 (7.1)	345 (6.8)	
**Impaired activity of daily living**^**d**^				0.36
Yes	1,428 (23.2)	238 (23.4)	1,190 (22.2)	
No	4,732 (76.8)	838 (76.6)	3,894 (77.8)	
**MMSE score, mean ± SD**	21.4 ± 9.2	21.5 ± 9.1	21.3 ± 9.2	0.46
**Cognitive impairment**^**e**^				0.60
With	1,537 (25.0)	270 (25.1)	1,267 (24.9)	
Without	4,623 (75.0)	806 (74.9)	3,817 (75.1)	
**Smoking**				0.25
Current	979 (15.9)	189 (17.5)	790 (15.5)	
Former	864 (14.0)	145 (13.5)	719 (14.1)	
Never	4,317 (70.1)	742 (69.0)	3,575 (70.3)	
**Alcohol consumption**				0.57
Binge drink	717 (11.6)	132 (12.3)	585 (11.5)	
Moderate drink	432 (7.0)	69 (6.4)	363 (7.1)	
Never drink	5,011 (81.3)	875 (81.3)	4,136 (81.4)	
**Regular physical activity**				0.97
Current	1,712 (27.8)	297 (27.6)	1,415 (27.8)	
Former	454 (7.4)	77 (7.2)	377 (7.4)	
Never	3,994 (64.8)	702 (65.2)	3,292 (64.8)	
**Dietary pattern**^**f**^				0.13
Unfavorable	1,817 (29.5)	292 (27.6)	1,525 (30.0)	
Intermediate	2,592 (41.5)	476 (44.2)	2,116 (41.6)	
Favorable	1,751 (28.4)	308 (28.6)	1,443 (28.4)	
**Weight (kg)**				0.56
<38	989 (16.1)	180 (16.7)	809 (15.9)	
38–50	2,960 (48.1)	507 (47.1)	2,453 (48.2)	
>50	2,211 (35.9)	389 (36.2)	1,822 (35.8)	
**Healthy lifestyle profile**^**g**^				0.49
Unhealthy	2,317 (37.6)	408 (37.9)	1,909 (37.5)	
Intermediate	2,617 (42.5)	461 (42.8)	2,156 (42.4)	
Healthy	1,226 (19.9)	207 (19.2)	1,019 (20.0)	

a Numbers shown are *N* (%) unless otherwise noted.

b ANOVA for continuous variables and chi-squared tests for categorical variables were applied to test the significance levels of the differences between *APOE* ɛ4 carriers and noncarriers.

c Manual worker includes professional, technical, governmental, institutional, or managerial personnel. Nonmanual worker includes those agriculture, forest, animal husbandry and fishery worker, industrial worker, commercial or service worker, military personnel, housework, and others.

d Activity of daily living: assessed by 6 self-reported questions: “Do you need assistance in bathing/dressing/toileting/transferring/eating/continence?”. Impaired activity of daily living was defined as if the participants answered “Yes” for any of those questions.

e Cognitive impairment: Cognitive impairment was defined by an MMSE score of less than 18.

f Dietary pattern: categorized by a simplified healthy eating index (unfavorable: 0–4; intermediate: 5–6; and favorable: 7–10).

g Healthy lifestyle profile: categorized by a healthy lifestyle score [healthy (8–10), intermediate (6–7), and unhealthy lifestyle (0–5)].

*APOE*, apolipoprotein E; IQR, interquartile range; MMSE, Mini-Mental State Examination; SD, standard deviation.

### Single lifestyle factors’ association with cognitive impairment

The associations of smoking, alcohol consumption, dietary pattern, physical activity, and body weight with cognitive impairment were presented in **Table 2**. Current physical activity and a favorable dietary pattern was significantly associated with lower odds of cognitive impairment compared with their counterparts after adjusting for a group of covariates (current versus never physical activity: odds ratio [OR]: 0.66, 95% confidence interval [CI]: 0.55, 0.79, *P* < 0.001; favorable versus unfavorable dietary pattern: OR: 0.42, 95% CI: 0.35, 0.51, *P* < 0.001). No significant association was found between smoking, alcohol consumption, body weight, and cognitive impairment in the adjusted model.

**Table 2:** Association between single lifestyle factor and cognitive impairment. *

Independent variable	Logistic regression, OR of cognitive impairment (95% CI)			
	Unadjusted model	*P* value	Adjusted model**	*P* value
**Smoking**				
Current	*Reference*		*Reference*	
Former	1.12 (0.85, 1.47)	0.43	0.98 (0.74, 1.31)	0.89
Never	1.18 (0.95, 1.49)	0.13	1.14 (0.90, 1.43)	0.51
**Alcohol consumption**				
Binge drinking	*Reference*		*Reference*	
Moderate drinking	0.73 (0.52, 1.03)	0.081	0.79 (0.55, 1.14)	0.21
Never drinking	1.14 (0.92, 1.41)	0.25	1.08 (0.86, 1.37)	0.90
**Physical activity**				
Never	*Reference*		*Reference*	
Former	1.13 (0.91, 1.41)	0.71	0.87 (0.66, 1.11)	0.25
Current	0.46 (0.40, 0.55)	<0.001	0.66 (0.55, 0.79)	<0.001
**Dietary pattern**				
Unfavorable	*Reference*		*Reference*	
Intermediate	0.69 (0.60, 0.79)	<0.001	0.66 (0.57, 0.77)	<0.001
Favorable	0.44 (0.38, 0.52)	<0.001	0.42 (0.35, 0.51)	<0.001
**Body weight (kg)**				
<38	*Reference*		*Reference*	
38–50	0.63 (0.54. 0.74)	<0.001	0.96 (0.80, 1.15)	0.59
>50	0.51 (0.44, 0.62)	<0.001	0.88 (0.70, 1.09)	0.21

* Cognitive impairment: Cognitive impairment was defined by an MMSE score of less than 18.

** Model adjusted for age, sex, residence, education level, marital status, *APOE* genotype, lifestyle factors (smoking, alcohol consumption, physical activity, body weight, and dietary pattern), activity of daily living, and 7 kinds of self-reported disease (COPD, tuberculosis, cancer, diabetes, hypertension, stroke, and cardiovascular disease).

*APOE*, apolipoprotein E; CI, confidence interval; COPD, chronic obstructive pulmonary disease; MMSE, Mini-Mental State Examination; OR, odds ratio.

### APOE ε4 genotype and lifestyle profiles’ associations with cognitive impairment

**Fig 1** presents the association between *APOE* genotypes, lifestyle profiles, and cognitive function. In the multivariable logistic regression, the *APOE* ε4 noncarriers had 17% lower odds of cognitive impairment (95% CI: 1% to 31%, *P* = 0.042) compared with those carrying the ε4 allele. Compared with those with an unhealthy lifestyle, participants with intermediate and healthy lifestyle were associated with 28% (95% CI: 16%, 38%, *P* < 0.001) and 55% (95% CI: 44%, 64%, *P* < 0.001) lower odds of cognitive impairment in the multivariable-adjusted model. In the longitudinal analysis, similar associations of cognitive decline with lifestyle profile and *APOE* genotypes were observed in the adjusted model (**S2 Table**) (healthy versus unhealthy lifestyle: OR: 0.72, 95% CI: 0.54, 0.95, *P* = 0.033; *APOE* ε4 noncarrier versus carrier: OR: 0.75, 95% CI: 0.60, 0.94, *P* = 0.027).

### Subgroup analyses and sensitivity analyses

**Fig 2:**
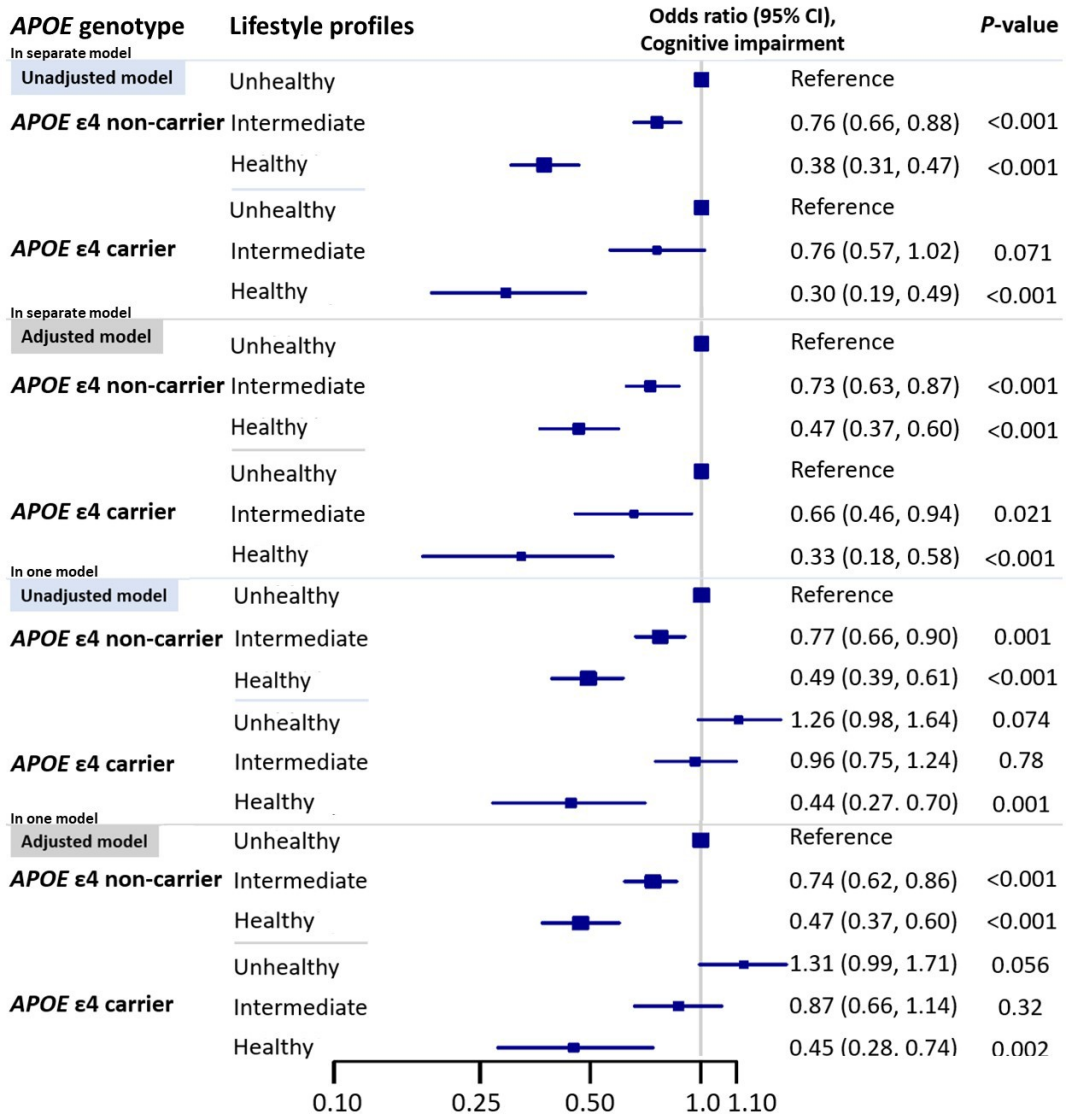
Associations* between cognitive impairment** and lifestyle profiles stratified by *APOE* genotype. *Models adjusted for age, sex, residence, education level, marital status, *APOE* genotype, activity of daily living, and 7 kinds of self-reported disease (COPD, tuberculosis, cancer, diabetes, hypertension, stroke, and cardiovascular disease). **Cognitive impairment: cognitive impairment was defined by an MMSE score of less than 18. *APOE*, apolipoprotein E; CI, confidence interval; COPD, chronic obstructive pulmonary disease; MMSE, Mini-Mental State Examination.

**Fig 2** presents the associations between lifestyle profiles and cognitive impairment stratified by *APOE* ε4 genotype. In the subgroup analyses by *APOE* genotype, among *APOE* ε4 noncarriers, the odds of cognitive impairment in participants with a healthy lifestyle was lower than that in those with an unhealthy lifestyle (OR: 0.47, 95% CI: 0.37 to 0.60, *P* < 0.001) (**Fig 2**). In the model among *APOE* ε4 carriers, the pattern was similar (panel B), and the interaction between *APOE* genotype and lifestyle profiles was not significant (*P*_*ε4 carriers x intermediate lifestyle*_ = 0.62, *P*_*ε4 carriers x healthy lifestyle*_ = 0.30). In the model including all participants and a variable indicating the joint classification of lifestyle profile and *APOE* ε4 genotype, we could observe the same dose–response relationship between lifestyle groups and cognitive impairment among both *APOE* ε4 carriers and noncarriers (**Fig 2**).

In supplemental analyses, we presented the associations between single lifestyle factors and cognitive impairment stratified by *APOE* genotype in **S1 Table**. The associations of cognitive impairment with smoking, alcohol consumption, physical activity, dietary pattern, and body weight did not vary by *APOE* genotype (all *P*s > 0.05).

Those sensitivity analyses showed that the associations between lifestyle profiles and cognitive impairment were consistent with our main analysis after (1) excluding deaths in 2 years after the baseline survey (*N* = 4,947, **S3 Table**); (2) excluding 122 participants with deafness and blindness (*N* = 6,038, **S4 Table**); (3) excluding 505 participants with an MMSE score equal to 0 (*N* = 5,655, **S5 Table**); (4) using BMI to build the healthy lifestyle score instead of body weight (*N* = 4107, **S6 Table**); (5) defining cognitive impairment with different MMSE cutoff scores (16, 21, and 25) and using the education-adjusted outcome (**S7 Table**); (6) adding blood pressure and diabetes in the lifestyle score (**S8 Table**); (7) using ordinal logistics model and mapping the MMSE score to CDR as outcome and using linear regression with MMSE score as outcome (**S9 Table**); and (8) using Poisson regression to estimate the risk ratios (**S10 Table**)

## Discussion

In this cross-sectional study of the Chinese oldest old (aged 80 years or older), we assessed the association between *APOE* ε4 genotype, lifestyle profiles, and cognitive impairment. Our results suggested that the odds of cognitive impairment was 17% (95% CI: 1% to 30%) lower among those *APOE* ε4 noncarriers (versus carriers) and 52% lower among those with healthy (versus unhealthy) lifestyle after controlling for sociodemographic, disability, and a number of chronic diseases. In addition, a healthier lifestyle was associated with lower odds of cognitive impairment regardless of *APOE* genotype.

Our study adds evidence to the relationships of gene and lifestyle with cognition among the oldest old. This population group are the fastest-growing segment of society and have a high risk of developing cognitive impairment with a prevalence up to 40% [30,31]. Such increasing number of older people with cognitive impairment and dementia have major clinical and financial consequences for patients, their families, and society [32]. However, effective interventions were reluctant to target on this special population group considering the benefit could be relatively small due to comparatively short expected life years than that among younger peers. In line with some of the previous studies that also found the lifestyle intervention could be beneficial for older adults in high-income countries [33], our study demonstrated that lifestyle intervention to the oldest old population could be beneficial for cognition throughout the whole life cycle, regardless of their *APOE* genotype. As the oldest old population group is rapidly growing in China as well as in other countries that have experienced population aging, cognitive outcomes in this age group may benefit from public health interventions including lifestyle modification programs.

Another defining feature of our study is that we examined lifestyle as a profile instead of single risk factors. Although there is growing evidence supporting the effect of individual lifestyle factors on cognition [13,34–36], few studies have explored the joint effects of healthy lifestyle profiles especially among the oldest old. Consistent with previous reports [8,9], the present analysis supported the hypothesis of a combined effect of healthy lifestyle on cognition and further extend this to the oldest old population. Of the 5 factors we examined, only 2 were found to be significant in multivariable models on single factors only. Nevertheless, the combined profile—defined based on prior studies [5,8–10]—was significantly associated with cognition, with healthier participants having less than half of the odds of cognitive impairment compared with the unhealthy ones. This result demonstrated the strengths of examining lifestyle factors as a group as they often cluster together and may create multiplicative instead of additive effects.

In our study, a significant association between *APOE* ε4 genotype and cognitive impairment was found, similar to a previous study among 425 Chinese elderly with an average age of 83 years [37]. However, another study among 1,445 Chinese elderly (average age: 71.95 ± 5.65) reported a lack of association between *APOE* genotype and cognitive function. One reason accounting for the inconsistency may be the differences in the ages of the study populations. In a study including 10,371 Koreans aged 45 to 74 years, carrying *APOE* ε4 allele was associated with lower MMSE scores only among those participants between 65 and 74 years old [38]. Additionally, a similar phenomenon was also found among the other races. In the study using the Cohorts for Heart and Aging Research in Genomic Epidemiology (CHARGE) Consortium data, which included 543,949 European participants from 31 cohorts, the magnitude of the association between rs10119 in the APOE region and worse cognitive ability increased with the mean age of each cohort. The effect was close to zero in younger cohorts, aged 55 to 60 years, and most pronounced in the oldest old [39]. Taken together, our finding of a positive association between *APOE* genotype and cognition among individuals 80 years or older may suggest that the effect of *APOE* genotype on cognitive function might be cumulative. To be more specific, with *APOE* ε4 allele’s negative influence on neuronal functioning, ε4 carriers may have a higher speed of neuronal cell loss which is irreversible [40]. Accordingly, during the early life stage, the difference in cognitive function between *APOE* ε4 carriers and noncarriers may not be significant, while as people age, *APOE* ε4 carriers may have a lower average cognitive function compared with noncarriers of the same age due to the cumulative damage of ε4 allele on neurons. On the other hand, in our study, ε4 noncarriers were slightly older than carriers, suggesting that there might be a survival effect from the ε4 genotype. If the more susceptible die earlier, there may be an attenuation of the relationships under study as people age. These complex relationships need to be further examined in future studies. Additionally, our findings suggest that the development of AD risk stratification tools may benefit from the incorporation of the role of age in considering the association between *APOE* genotype and cognition.

The interaction between *APOE* genotype and individual lifestyle factors or lifestyle as a profile is controversial [11–13,23,24]. In a study with 14 years of follow-up and an average age at baseline of 69 years old [8], healthy lifestyle profiles only showed its benefits among *APOE* ε4 noncarriers. Yet another study from the UK Biobank (*N* = 196,383 average age: 64.1 years and median follow-up 8.2 years) found that favorable lifestyle profile was related to lower dementia risk regardless of the genetic dementia risk measured by a polygenetic risk score including *APOE* genotype [9]. Consistent with the UK Biobank study, we found that the association between lifestyle profiles and cognitive function did not vary by *APOE* ε4 genotype. These findings provide an optimistic perspective that a healthy lifestyle was still associated with lower odds of cognitive impairment among those with high genetic dementia risk. Our results suggest that although genetic risk cannot be modified from the genome, modifiable lifestyle factors are associated with cognitive outcomes independent of genetic risk even at a very advanced age. It may be beneficial for physicians to recommend that the oldest old adopt a healthier lifestyle to improve cognitive outcomes regardless of current cognitive function and genetic AD risk. Additionally, our results, if further validated by longitudinal studies among the oldest old population, may suggest that lifestyle interventions among those oldest old with high genetic risk may be still effective in promoting better cognitive performance.

Methodologic strengths of this study include a large sample size of the oldest old and the combination of 5 lifestyle factors instead of single factors. Our study also has several limitations: (1) it has a cross-sectional design and cannot evaluate changes in lifestyles or establish causality. However, our results were robust to adjustment of a number of indicators such as diseases and activity of daily living. Nevertheless, prospective studies on the incidence of cognitive impairment are warranted; (2) the lifestyle measurements were self-reported thus nonspecific, and some measurements such as total cholesterol or high-density lipoproteins included in the established lifestyle metrics were not available in CLHLS [41]. However, the categorization based on the summary score has been shown to be a simple and powerful tool to classify people into different lifestyle groups, also those 5 factors were regarded as common behavior factors; (3) we used the Chinese version of MMSE to measure cognition, which is not a clinical diagnosis for cognitive impairment [42,43]. However, it is a validated instrument in population-based studies; (4) the CI of the association between *APOE* genotype and cognitive impairment was wide. But, its association was demonstrated in prior studies with large sample size [44]. Of note, the magnitude of the association between *APOE* ε4 genotype and cognition in our study was low (about 20% increased odds) compared to some other studies that found up to 3-fold higher risks [45]. Although our finding of the low magnitude was consistent with other studies conducted among adults 80 years and older [46], this rather weak association could partly explain why a healthy lifestyle “outweighed” genetic makeup in relation to cognitive impairment; and (5) lastly, because our study sample only came from the oldest old in China from the CLHLS with their unique characteristics such as low body weight compared to populations of high-income countries, its generalizability may be limited.

## Conclusions

In summary, we found that the *APOE* genotype and lifestyle profiles were independently associated with cognitive impairment. In addition, the association between lifestyle profile and cognition was independent of *APOE* genotype among Chinese oldest old. Our results, corroborated by other interventional studies on lifestyle modification and cognitive function [47], support the importance of maintaining healthy lifestyle throughout the life course, even among the oldest old.

## Supporting information

S1 Checklist STROBE checklist. STROBE, Strengthening the Reporting of Observational Studies in Epidemiology.
(DOCX)

S1 Text Methods section for the sensitivity analysis. (DOCX)

S2 Text Development of the statistical analysis plan. (DOCX)

S3 Text Copy of the survey questions (used variables marked as red). (DOC)

S1 Fig The adjusted OR of cognitive impairment of body weight and BMI in logistics regression models with penalized splines. Adjustment: age at baseline, sex, residency, education level, *APOE* genotype, lifestyle factors (smoking, alcohol consumption, dietary pattern, and physical activity), activity of daily living, and 7 kinds of self-reported disease (COPD, tuberculosis, all-cause cancer, diabetes, hypertension, stroke, and cardiovascular disease). The logistics regression models with penalized splines evaluated nonlinear associations of cognitive impairment with weight and BMI; 2 cutoffs were identified (weight: less than 38 kg or higher than 50 kg, BMI: lower than 18 kg/m^2^ or higher than 21 kg/m^2^) above and below, in which there was no significant increase in the multitude of OR for cognitive impairment. *APOE*, apolipoprotein E; BMI, body mass index; COPD, chronic obstructive pulmonary disease; OR, odds ratio.
(DOCX)

S2 Fig The adjusted OR of cognitive impairment for lifestyle score and modifiable factor score in logistics regression models with penalized splines. Adjustment: age at baseline, sex, residency, education level, *APOE* genotype, activity of daily living, and 7 kinds of self-reported disease (COPD, tuberculosis, all-cause cancer, diabetes, hypertension, stroke, and cardiovascular disease). The logistics regression models with penalized splines evaluated nonlinear associations of cognitive impairment with lifestyle score and modifiable factor score; 2 cutoffs were identified (lifestyle score: less than 6 or higher than 7; modifiable factor score: less than 5 or higher than 8) above and below, in which there was no significant increase in the multitude of OR for cognitive impairment. *APOE*, apolipoprotein E; COPD, chronic obstructive pulmonary disease; OR, odds ratio.
(DOCX)

S1 Table Association between single lifestyle factors and cognitive impairment stratified by *APOE* genotype. Cognitive impairment: Cognitive impairment was defined by MMSE scores less than 18. Adjustment: age at baseline, sex, residency, education level, *APOE* genotype, activity of daily living, and 7 kinds of self-reported disease (COPD, tuberculosis, all-cause cancer, diabetes, hypertension, stroke, and cardiovascular disease). *APOE*, apolipoprotein E; COPD, chronic obstructive pulmonary disease; MMSE, Mini-Mental State Examination.
(DOCX)

S2 Table Associations of cognitive decline with *APOE* ε4 genotype and lifestyle profiles (*N* = 3,136 with 1,564 decline). Model was adjusted for age at baseline, baseline MMSE score, sex, residency, education level, marital status, *APOE* genotype, lifestyle profile, activity of daily living, and 7 kinds of self-reported disease (COPD, tuberculosis, all-cause cancer, diabetes, hypertension, stroke, and cardiovascular disease). *APOE*, apolipoprotein E; COPD, chronic obstructive pulmonary disease; MMSE, Mini-Mental State Examination.
(DOCX)

S3 Table Sensitivity analysis: associations of cognitive function with *APOE* ε4 genotype and lifestyle profiles excluding the participants dead in 2 years after baseline survey (*N* = 4,947). Model was adjusted for age at baseline, sex, residency, education level, marital status, *APOE* genotype, lifestyle profile, activity of daily living, and 7 kinds of self-reported disease (COPD, tuberculosis, all-cause cancer, diabetes, hypertension, stroke, and cardiovascular disease). *APOE*, apolipoprotein E; COPD, chronic obstructive pulmonary disease.
(DOCX)

S4 Table Sensitivity analysis: associations of cognitive function with *APOE* ε4 genotype and lifestyle profiles excluding the participants with deafness or blindness (*N* = 6,038). Model was adjusted for age at baseline, sex, residency, education level, marital status, *APOE* genotype, lifestyle profile, activity of daily living, and 7 kinds of self-reported disease (COPD, tuberculosis, all-cause cancer, diabetes, hypertension, stroke, and cardiovascular disease). *APOE*, apolipoprotein E; COPD, chronic obstructive pulmonary disease.
(DOCX)

S5 Table Sensitivity analysis: associations of cognitive function with *APOE* ε4 genotype and lifestyle profiles excluding the participants with MMSE score equal to 0 (*N* = 5,655). Model was adjusted for age at baseline, sex, residency, education level, marital status, *APOE* genotype, lifestyle profile, activity of daily living, and 7 kinds of self-reported disease (COPD, tuberculosis, all-cause cancer, diabetes, hypertension, stroke and cardiovascular disease). *APOE*, apolipoprotein E; COPD, chronic obstructive pulmonary disease; MMSE, Mini-Mental State Examination.
(DOCX)

S6 Table Sensitivity analysis: associations of cognitive function with *APOE* ε4 genotype and lifestyle profiles using BMI instead of body weight to build the healthy lifestyle score (*N* = 4,126). Model was adjusted for age at baseline, sex, residency, education level, marital status, *APOE* genotype, lifestyle profile, activity of daily living, and 7 kinds of self-reported disease (COPD, tuberculosis, all-cause cancer, diabetes, hypertension, stroke, and cardiovascular disease). *APOE*, apolipoprotein E; BMI, body mass index; COPD, chronic obstructive pulmonary disease.
(DOCX)

S7 Table Sensitivity analysis: associations of cognitive function with *APOE* ε4 genotype and lifestyle profiles defining cognitive impairment with different MMSE cutoff and using the education-adjusted outcome. Model was adjusted for age at baseline, sex, residency, education level, marital status, *APOE* genotype, lifestyle profile, activity of daily living, and 7 kinds of self-reported disease (COPD, tuberculosis, all-cause cancer, diabetes, hypertension, stroke, and cardiovascular disease). *APOE*, apolipoprotein E; COPD, chronic obstructive pulmonary disease; MMSE, Mini-Mental State Examination.
(DOCX)

S8 Table Sensitivity analysis: associations of cognitive function with *APOE* ε4 genotype and lifestyle profiles by adding blood pressure and diabetes in the lifestyle score. Model was adjusted for age at baseline, sex, residency, education level, marital status, *APOE* genotype, lifestyle profile, activity of daily living, and 7 kinds of self-reported disease (COPD, tuberculosis, all-cause cancer, diabetes, hypertension, stroke, and cardiovascular disease). *APOE*, apolipoprotein E; COPD, chronic obstructive pulmonary disease.
(DOCX)

S9 Table Sensitivity analysis: associations of cognitive function with *APOE* ε4 genotype and lifestyle profiles using ordinal logistics model and mapping the MMSE score to CDR as outcome/using linear regression with MMSE score as outcome. Model was adjusted for age at baseline, sex, residency, education level, marital status, *APOE* genotype, lifestyle profile, activity of daily living, and 7 kinds of self-reported disease (COPD, tuberculosis, all-cause cancer, diabetes, hypertension, stroke, and cardiovascular disease). *APOE*, apolipoprotein E; CDR, Clinical Dementia Rating; COPD, chronic obstructive pulmonary disease; MMSE, Mini-Mental State Examination.
(DOCX)

S10 Table Sensitivity analysis: associations of cognitive function with *APOE* ε4 genotype and lifestyle profiles using Passion regression. Model was adjusted for age at baseline, sex, residency, education level, marital status, *APOE* genotype, lifestyle profile, activity of daily living, and 7 kinds of self-reported disease (COPD, tuberculosis, all-cause cancer, diabetes, hypertension, stroke, and cardiovascular disease). *APOE*, apolipoprotein E; COPD, chronic obstructive pulmonary disease.
(DOCX)

## Abbreviations

AD: Alzheimer disease
APOE: apolipoprotein E
BGI: Beijing Genomics Institute
BMI: body mass index
CDR: Clinical Dementia Rating
CHARGE: Cohorts for Heart and Aging Research in Genomic Epidemiology
CI: confidence interval
CLHLS: Chinese Longitudinal Healthy Longevity Survey
COPD: chronic obstructive pulmonary disease
HR: hazard ratio
MMSE: Mini-Mental State Examination
OR: odds ratio
SD: standard deviation
STROBE: Strengthening the Reporting of Observational Studies in Epidemiology
