# Supplementary material for: Association of APOE ε4 genotype and lifestyle with cognitive function among Chinese adults aged 80 years and older: A cross-sectional study
Source: PLoS Med. 2021 Jun 1;18(6):e1003597. doi: 10.1371/journal.pmed.1003597 (PMC8168868; doi:10.1371/journal.pmed.1003597)
Supplement: S1 Text — (DOCX) [file pmed.1003597.s002.docx]

**S1 Text. Method section for the sensitivity analysis**

**Cognitive decline**

Cognitive decline was assessed using the rate of change in MMSE score, which was calculated as the difference between the MMSE score at baseline and the second survey divided by the number of years between the two examinations ((MMSE score at baseline − MMSE score at the second survey)/the interval between two examinations, years). We then categorized the rate of change in the MMSE score into two groups: cognitive decline (a rate of change in MMSE score less than zero), stable cognitive function (a rate of change in MMSE score equal to zero or higher). We excluded 5% participants with the highest or lowest 2.5% cognitive decline rate (N=157) as the change of MMSE score was higher than 20 which might be caused by the measurements error. Totally, 3,136 participants were included in the longitudinal analysis

The association between healthy lifestyle, *APOE* phenotype and cognitive decline was assessed using multivariable logistic regression. The model adjusted for age, sex, residency, education level and marital status, activity of daily living and seven kinds of self-reported diseases (chronic obstructive pulmonary disease (COPD), tuberculosis, all-cause cancer, diabetes, hypertension, stroke and cardiovascular disease).

**Modifiable risk factor score**

In some other established lifestyle scores such as Cardiovascular Health metric [1], or Pooled Cohort Equations [2], the information of blood pressure and diabetes were included. In addition, high blood pressure and diabetes were the risk factors of cognitive impairment [3, 4]. To make our lifestyle score more replicable, we additionally added the hypertension and diabetes into the lifestyle score and make it a modifiable risk factor score ranged from 0 to 15, with higher score indicate better modifiable profile.

After participants had rested for at least five minutes, research assistants took two measurements of blood pressure on the right arm by mercury sphygmomanometer (upper arm type; Yuyue, Jiangsu, China) at the heart level. For bedridden participants, blood pressure measurements were obtained in a recumbent position. The mercury sphygmomanometer must be calibrated before each measurement. Systolic blood pressure (SBP) and diastolic blood pressure (DBP) were calculated as the average of the two measurements taken for an individual. We defined hypertension as (1) systolic blood pressure (SBP) ≥140 millimeters of mercury (mm Hg), a diastolic blood pressure (DBP) ≥90 mm Hg; (2) Current treatment with anti-hypertensive drugs in participants with a self-reported history of diagnosed hypertension. Severe hypertension was defined as SBP ≥180 mm Hg or DBP ≥120 mm Hg. “Without hypertension”, “hypertension” and “severe hypertension” received a score of 2, 1, or 0 respectively with higher scores indicated healthier blood pressure status. The information of diabetes was assessed by a three terms question “not have diabetes”, ”have diabetes, but it did not influence the daily life”, ”have diabetes and it influence the daily life”, those three terms received a score of 2, 1, 0 respectively.

**Reference**

1. Rana JS, Liu JY, Moffet HH, Karter AJ, Nasir K, Solomon MD, et al. Risk of atherosclerotic cardiovascular disease by cardiovascular health metric categories in approximately 1 million patients. Eur J Prev Cardiol. 2020:2047487320905025. Epub 2020/02/11. doi: 10.1177/2047487320905025. pmid: 32039636

2. Preiss D, Kristensen SL. The new pooled cohort equations risk calculator. Can J Cardiol. 2015;31(5):613-9. Epub 2015/04/07. doi: 10.1016/j.cjca.2015.02.001. pmid: 25843167

3. Gregg EW, Yaffe K, Cauley JA, Rolka DB, Blackwell TL, Narayan KM, et al. Is diabetes associated with cognitive impairment and cognitive decline among older women? Study of Osteoporotic Fractures Research Group. Arch Intern Med. 2000;160(2):174-80. Epub 2000/01/27. doi: 10.1001/archinte.160.2.174. pmid: 10647755

4. Goldstein FC, Levey AI, Steenland NK. High blood pressure and cognitive decline in mild cognitive impairment. J Am Geriatr Soc. 2013;61(1):67-73. Epub 2013/01/11. doi: 10.1111/jgs.12067. pmid: 23301925
