# Supplementary material for: Association of APOE ε4 genotype and lifestyle with cognitive function among Chinese adults aged 80 years and older: A cross-sectional study
Source: PLoS Med. 2021 Jun 1;18(6):e1003597. doi: 10.1371/journal.pmed.1003597 (PMC8168868; doi:10.1371/journal.pmed.1003597)
Supplement: S2 Text — (DOCX) [file pmed.1003597.s003.docx]

**S2 Text. Development of the Statistical Analysis Plan**

Study Context

Data for the present study come from the CLHLS study, , a longitudinal study since 1998 with follow-up surveys every 2 to 3 years. The CLHLS randomly selected 806 cities and counties in 23 provinces of China using multi-stage stratified sampling. The study areas covered 85% Chinese population. More details on sampling design and data quality can be found elsewhere. The design of CLHLS was approved by the Campus Institutional Review Board of Duke University (Pro00062871) and the Biomedical Ethics Committee of Peking University (IRB00001052-13074). All participants or their legal representatives signed written consent forms during the surveys.

The present study is a cross-sectional observational analysis that was conceptualized after the baseline data collection was completed. This idea of this study was driven from two recent papers from JAMA [1] and Nature Medicine (NM) [2]. Those two studies reported inconsistent results of the interaction between genetic risk and lifestyle profiles on incidence of dementia. The JAMA paper reported that there is no interaction between lifestyle and genetic dementia risk while the NM paper reported that the association between lifestyle and dementia was only significant among those *APOE* ε4 non-carrier. The authors provided a potential explanation that the higher average age of the NM’s sample may cause this discrepancy (64.1 vs. 69.1 years old). We thought a good way to test this hypothesis would be to examine its interaction among those aged over 80 years old. CLHLS study could provide suitable data because the mean age of the sample was high and there is a genetic sub-study which provided the information of *APOE* genotype. Thus, we conducted this study.

Analysis Plan prior to commencing the present manuscript

The analyses presented in the current manuscript evolved from the following research question and associated statistical analysis:

1. *What is the association between APOE gene and cognitive function among oldest old?*
   1. *We will use the pooled baseline cross-sectional data from CLHLS genetic sub-study.*
   2. *Cognitive impairment will be the primary outcome for this analysis defined by MMSE lower than 18.*
   3. *The APOE genotype will be group by whether the participants carrying APOE ε4 allele (ε2/ε4, ε3/ε4, ε4/ε4 genotypes) or not (ε2/ε2, ε2/ε3, ε3/ε3)*
2. *What is the association between lifestyle profile and cognitive function?*
   1. *A lifestyle score will be built by five finds of behavior lifestyle factors- smoking, drinking, dietary pattern, physical activity and body weight. BMI will be used in the sensitivity analysis. The score will be built by summing those single lifestyle factors*
   2. *We will use the logistics regression model to evaluate the association of cognition with lifestyle profile and APOE genotype*
3. *What is the interaction between lifestyle profile and APOE genotype on cognition*
   1. *Examine the effect modification of APOE genotype on the association of lifestyle and cognition by subgroup analysis*

Changes to the Analysis Plan after beginning analysis for the present manuscript

Based on the feedback from co-authors and the study team meetings, the following revisions to the analysis plan were made:

1. Using the 3-terms single lifestyle factors to build the score, eg, Dietary pattern: favorable:2; intermediate:1; unfavorable:0
2. Adding the association of single lifestyle factor with cognition in the main results
3. Adding analysis of the interaction between single lifestyle factor and *APOE* genotype
4. Sensitivity analysis: excluding those who dead in two years after the baseline survey

Based on the feedback from reviewers, the following revisions to the analysis plan were made:

1. Sensitivity analyses: using longitudinal cognitive decline as outcome
2. Sensitivity analyses: using different MMSE cut-off scores (lower than 16, 21 or 25) to define cognitive impairment and adopting different MMSE cutoff scores based on education level to define cognitive impairment (<18 for those without formal education, <21 for those with 1-6 years of education and <25 for those with more than six years of education)
3. Sensitivity analyses: incorporating blood pressure and diabetes into the lifestyle score to build a modifiable  factor score and using this score to reproduce the analysis
4. Sensitivity analyses: mapping the MMSE score to Clinical Dementia Rating (CDR) and using the CDR as outcome and ordinal logistics model to reproduce the analysis for increasing the statistical power
5. Sensitivity analyses: excluding the participants with deafness or blindness
6. Sensitivity analyses: repeating the analyses using Poisson regression models

**Reference**

1. Lourida I, Hannon E, Littlejohns TJ, Langa KM, Hypponen E, Kuzma E, et al. Association of Lifestyle and Genetic Risk With Incidence of Dementia. JAMA. 2019. Epub 2019/07/16. doi: 10.1001/jama.2019.9879. pmid: 31302669
2. Licher S, Ahmad S, Karamujic-Comic H, Voortman T, Leening MJG, Ikram MA, et al. Genetic predisposition, modifiable-risk-factor profile and long-term dementia risk in the general population. Nat Med. 2019;25(9):1364-9. Epub 2019/08/28. doi: 10.1038/s41591-019-0547-7. pmid: 31451782
