# Supplementary material for: Association of APOE ε4 genotype and lifestyle with cognitive function among Chinese adults aged 80 years and older: A cross-sectional study
Source: PLoS Med. 2021 Jun 1;18(6):e1003597. doi: 10.1371/journal.pmed.1003597 (PMC8168868; doi:10.1371/journal.pmed.1003597)
Supplement: S3 Text — (DOC) [file pmed.1003597.s004.doc]

**S3 Text. Copy of the survey questions (Used variables marked as red)**

## I. category of the interviewee:

1. elder who was interviewed in 1998; 2. newly added elder to replace the deceased elder; 3. newly became centenarians or became 80 or 81 years in 2000; 4. sibling of 1, 2, or 3, and aged 80+.

| *if answer 1, 2, or 3, please jump to III* |
| --- |

## II. If (s)he is a sibling of interviewed elder, what kind of sibling

1. sibling 2. half sibling 3. adopted sibling 4. others

III. current residence area of interviewee

1. city 2. town 3. rural

IV. validated age

| **A. Basic Information** | |  | Code |
| --- | --- | --- | --- |
| A1 Sex | 1 male 2 female |  |  |
| A2 Ethnic group | ________ |  |  |
| A3.1 Animal year of interviewee’s birth | 1 rat 2 ox 3 tiger 4 rabbit  5 dragon 6 snake 7 horse  8 sheep 9 monkey 10 rooster  11 dog 12 boar |  |  |
| A3.2 Date of birth: |  |  |  |
| 1. western calendar 2. Chinese calendar | year________ month____  year________ month____ |  |  |
| A4.1 Which province were you born? | province __________ |  |  |
| A4.2 Which county (city) were you born? | 1. same as current address 2. other city or county |  |  |
| A4.3 Was the place of birth an urban area or a rural area (at time of birth)? | 1 urban 2 rural |  |  |
| A5.1 Co-residence | 1 with household member(s)  2 alone---*jump to A5.4*  3 in a nursing home---*jump to A5.4* |  |  |
| A5.2 How many people are living with you? | _______ person(s) |  |  |

| A5.3 Who are they?  *(Use back of paper for additional people if necessary.)*  *Note:*  *If ages are unknown, please fill in ‘888’.*  Relationship with interviewee:  0 spouse  1 child  2 spouse of child  3 grandchild  4 spouse of grandchild  5 great grandchild or spouse of great grandchild  6 sibling  7 parent or parent-in-law  8 other _____________ | relationship with interviewee | name | sex | age |  | relation-ship | age | sex |
| --- | --- | --- | --- | --- | --- | --- | --- | --- |
|  |  |  |  |  |  |  |  |  |
|  |  |  |  |  |  |  |  |  |
|  |  |  |  |  |  |  |  |  |
|  |  |  |  |  |  |  |  |  |
|  |  |  |  |  |  |  |  |  |
|  |  |  |  |  |  |  |  |  |
|  |  |  |  |  |  |  |  |  |
|  |  |  |  |  |  |  |  |  |
|  |  |  |  |  |  |  |  |  |
|  |  |  |  |  |  |  |  |  |
|  |  |  |  |  |  |  |  |  |
|  |  |  |  |  |  |  |  |  |
| Jump to Part B | | | | | | | | |
| A5.4 If living alone or in nursing home, since when? | year________ month _______ | | | |  |  | | |

| **B. Life Evaluation and Personality (to be answered only by interviewee)** | | | Code |
| --- | --- | --- | --- |
| * B1.1 How do you rate your life at present? | 1 very good 2 good  3 so so 4 bad  5 very bad  8 not able to answer |  |  |
| * B1.2 How do you rate your health at present? | 1 very good 2 good  3 so so 4 bad  5 very bad  8 not able to answer |  |  |
| * B2.1 Do you always look on the bright side of things? | 1 always 2 often 3 sometimes 4 seldom 5 never  8 not able to answer |  |  |
| * B2.2 Do you like to keep your belongings neat and clean? | 1 always 2 often 3 sometimes 4 seldom 5 never  8 not able to answer |  |  |
| * B2.3 Do you often feel fearful or anxious? | 1 always 2 often 3 sometimes 4 seldom 5 never  8 not able to answer |  |  |
| * B2.4 Do you often feel lonely and isolated? | 1 always 2 often 3 sometimes 4 seldom 5 never  8 not able to answer |  |  |
| * B2.5 Can you make your own decisions concerning your personal affairs? | 1 always 2 often 3 sometimes 4 seldom 5 never  8 not able to answer |  |  |
| * B2.6 Do you feel the older you get, the more useless? | 1 always 2 often 3 sometimes 4 seldom 5 never  8 not able to answer |  |  |
| * B2.7 Are you as happy as when you were younger? | 1 always 2 often 3 sometimes 4 seldom 5 never  8 not able to answer |  |  |

| **C. Mini Mental State Examination (MMSE)** | |  | Code |
| --- | --- | --- | --- |
| C1 ORIENTATION (to be answered only by interviewee) | |  |  |
| * C1.1 What time of day is it right now (morning, afternoon, evening)? | 1 correct 0 wrong  8 not able to answer |  |  |
| * C1.2 What is the month (Western or Chinese calendar) right now? | 1 correct 0 wrong  8 not able to answer |  |  |
| * C1.3 What is the date (day and month) of the mid-autumn festival? | 1 correct 0 wrong  8 not able to answer |  |  |
| * C1.4 What is the season right now? | 1 correct 0 wrong  8 not able to answer |  |  |
| * C1.5 What is the name of this county or district? | 1 correct 0 wrong  8 not able to answer |  |  |
| * C1.6 Please name as many kinds of food as possible in 1 minute. | _______ (kinds of food) |  |  |
| C2 REGISTRATION (to be answered only by interviewee) | |  |  |
| * C2.1 I am now going to test your memory. I will mention three objects.  (*Mention the following three objects without making a pause:)*  table, apple, cloth  Please repeat these three objects.  *(Evaluation based on first attempt only).*  *---- If all three questions are answered correct, please jump to C3.1*  table  apple  cloth | 1 correct 0 wrong  8 not able to answer  _______  _______  _______ |  |  |
|
| *C2.2 *If answers are insufficient or incorrect on first attempt, repeat the names of all objects until the interviewee is able to name all three of them (6 attempts at maximum). Write the number of attempts* *(e.g. ‘1’, if all three objects are repeated correctly on first attempt). Write ‘7’ if interviewee cannot repeat the names even after 6 attempts.* | ________attempts |  |  |
| C3 ATTENTION AND CALCULATION (to be answered only by interviewee) | | |  |
| * C3.1 I will ask you to spend 3 dollars from 20 dollars, then you must spend 3 dollars from the number you arrived at and continue to spend 3 dollars until you are asked to stop. |  |  |  |
| *(Circle ‘1’ each time the difference is 3 – even if a former answer was wrong. If the interviewee forgets the last number, the interviewer should repeat it, but then circle ‘0’ even if the answer was correct. Maximum score = 5 correct answers.)* | 1^st^ 1 correct 0 wrong  8 not able to answer  2^nd^ 1 correct 0 wrong  8 not able to answer  3^rd^ 1 correct 0 wrong  8 not able to answer  4^th^ 1 correct 0 wrong  8 not able to answer  5^th^ 1 correct 0 wrong  8 not able to answer |  |  |
| * C3.2 Ask the interviewee to draw the figure on Card B.  *(Circle ‘1’ if all the sides and angles are correct and if the figure in the middle is a quadrangle. Otherwise, circle ‘0’.)* | 1 correct 0 wrong  8 not able to do this |  |  |
| C4 RECALL (to be answered only by interviewee) | |  |  |
| * C4.1 Please repeat the three words (in any order) that I asked you to repeat a little while ago.  (Note the correct or the wrong answers as the scores.)  table  apple  cloth | 1 correct 0 wrong  8 not able to do this  ______  ______  ______ |  |  |
| C5 LANGUAGE (to be answered only by interviewee) | |  |  |
| * C5.1 *Give the interviewee a pen and then a watch and ask what these objects are called (1 point for each correct answer).*  Pen  Watch | 1 correct 0 wrong  8 not able to answer  _______  _______ |  |  |
| * C5.2 I will now ask you to repeat the following sentence:  ‘What you plant, what you will get.’  *(Circle ‘1’ only if repeated correctly on the first attempt.)* | 1 correct 0 wrong  8 not able to answer |  |  |
| * C5.3 I will give you a piece of paper. You must take the paper using your right hand, fold it in the middle using both hands, and place the paper on the floor*.*  *(Read the text aloud and hand a piece of paper to the interviewee. Give the paper directly to the interviewee. Do not repeat the instructions and do not offer any help. Note every movement as correct if it is made in the correct order.)*  right hand  folding  on the floor | 1 correct 0 wrong  8 not able to do  _______  _______  _______ |  |  |
| C5.4 Was the interviewee able to answer the questions in sections B and C? (answered *by Interviewer ONLY*) | 1 yes 2 no 3 partly |  |  |
| C5.5 If ‘no’ or ‘partly’, what is the main reason?  (answered *by Interviewer ONLY*) | 1 visually impaired, but can hear  2 hearing impaired, but can see  3 visually and hearing impaired  4 paralyzed  5 did not wish to participate  6 could not understand because of cognitive impairment  7 not able to participate at the moment because of some temporary illness such as a cold  8 other (please explain):  ________________________ |  |  |

| **D. LIFE STYLE** | | | | | | | Code | | | |
| --- | --- | --- | --- | --- | --- | --- | --- | --- | --- | --- |
| D1 Please tell us the main food you eat. | | 1 rice  2 corn (maize)  3 wheat (noodles and bread etc.)  4 other:_______________ | | | |  |  | | | |
| D2 How much of the above food do you normally eat per day? | | ________ liang | | | |  |  | | | |
| D3.1 Do you eat fresh fruit? | | 1 almost everyday year round  2 almost everyday except in winter  3 occasionally  4 rarely or never | | | |  |  | | | |
| D3.2 Do you eat fresh vegetables? | | 1 almost everyday year round  2 almost everyday except in winter  3 occasionally  4 rarely or never | | | |  |  | | | |
| D4 Please tell me what other kinds of food you normally eat and how often. | | around age 60 | | at present | |  | around age 60 | | at present | |
| 1 almost everyday  2 occasionally  3 rarely or never | Meat |  | |  | |  |  | |  | |
|  | Fish |  | |  | |  |  | |  | |
|  | Eggs |  | |  | |  |  | |  | |
|  | Food made from beans (tofu, etc.) |  | |  | |  |  | |  | |
|  | Salt-preserved vegetables |  | |  | |  |  | |  | |
|  | Sugar |  | |  | |  |  | |  | |
|  | Tea |  | |  | |  |  | |  | |
|  | Garlic |  | |  | |  |  | |  | |
| D5 What kind of water do you usually drink? | | 1 boiled water 2 un-boiled water | | | |  |  | | | |
| D6 Such water is (was): | | childhood | around age 60 | | at present |  | childhood | age 60 | | present |
| 1 water from a well  2 water from a river or lake  3 water from a spring  4 water from a pond or pool  5 tap water | |  |  | |  |  |  |  | |  |
| D7.1 Do you smoke at the present time? | | 1 yes 2 no | | | |  |  | | | |
| D7.2 Did you smoke in the past? | | 1 yes 2 no | | | |  |  | | | |
| If the answers of D7.1 and D7.2 are both ‘no’, please jump to D8.1 | | | | | | | | | | |
| D7.3 How old were you when you began to smoke? | | age _______ | | | |  |  | | | |
| D7.4 How old were you when you stopped smoking if you don’t smoke at present? | | age _______ | | | |  |  | | | |
| D7.5 If you smoke at the present time (or smoked in the past), how many times per day on average do you smoke? | | times _______ | | | |  |  | | | |
| D8.1 Do you drink alcohol at the present time? | | 1 yes 2 no | | | |  |  | | | |
| D8.2 Did you drink alcohol in the past? | | 1 yes 2 no | | | |  |  | | | |
| If the answers of D8.1 and D8.2 are both ‘no’, please jump to D9.1 | | | | | | | | | | |
| D8.3 How old were you when you began to drink alcohol? | | age _______ | | | |  |  | | | |
| D8.4 How old were you when stopped drinking alcohol if you don’t drink alcohol at present? | | age _______ | | | |  |  | | | |
| D8.5 If you drink at the present time (or drank in the past) , what kind of alcohol do you drink? | | 1 very strong liquor (≥38^0^)  2 not very strong liquor (<38^0^)  3 wine 4 rice wine 5 beer 6 others | | | |  |  | | | |
| D8.6 If you drink alcohol at the present time (drank in the past), how much per day on average do you drink? | | _______liang | | | |  |  | | | |
| D9.1 Do you do exercises regularly at present? | | 1 yes 2 no | | | |  |  | | | |
| D9.2 Did you do exercises regularly in the past? | | 1 yes 2 no | | | |  |  | | | |
| If the answers of D9.1 and D9.2 are both ‘no’, please jump to D10.1 | | | | | | | | | | |
| D9.3 How old were you when you began to do exercises? | | age _______ | | | |  |  | | | |
| D9.4 How old were you when you stopped doing exercises if you don’t do exercises at present? | | age _______ | | | |  |  | | | |
| D10.1 Have you done physical labour regularly? | | 1 yes 2 no---*jump to D11* | | | |  |  | | | |
| D10.2 If yes, from which age | | from age _______ | | | |  |  | | | |
| D10.3 to which age? | | to age _______ | | | |  |  | | | |

| D11 Do you now perform the following activities regularly? |  |  |  |
| --- | --- | --- | --- |
| Housework | 1 almost everyday 2 sometimes  3 never |  |  |
| grow vegetables & other field work | 1 almost everyday 2 sometimes  3 never |  |  |
| garden work | 1 almost everyday 2 sometimes  3 never |  |  |
| read newspapers/books | 1 almost everyday 2 sometimes  3 never |  |  |
| raise domestic animals | 1 almost everyday 2 sometimes  3 never |  |  |
| play cards and/or mah-jong | 1 almost everyday 2 sometimes  3 never |  |  |
| watch TV and/or listen to radio | 1 almost everyday 2 sometimes  3 never |  |  |
| religious activities | 1 almost everyday 2 sometimes  3 never |  |  |

| E. KATZ' ADL  For each area of functioning listed below, check the description that applies. (The word ‘assistance’ means supervision, direction, or personal assistance.) | | | Code |
| --- | --- | --- | --- |
| E1 **Bathing** – either sponge bath, tub bath, shower or washing the body | 1 receives no assistance (gets in and out of tub alone if tub is usual means of bathing)  2 receives assistance in bathing only for part of the body (such as back or a leg)  3 receives assistance in bathing more than one part of the body (or doesn’t bathe) |  |  |
| E2 **Dressing –** gets clothes from closets and drawers – including underwear, outer garments and fasteners (including suspenders if worn ) | 1 gets clothes and gets completely dressed without assistance  2 gets clothes and gets dressed without assistance except for tying shoes  3 receives assistance in getting clothes or in getting dressed, or stays partly or completely undressed |  |  |
| E3 **Toilet –** going to the toilet; cleaning oneself afterwards | 1 goes to the toilet, cleans self, and arranges clothes without assistance (may use object for support such as cane, walker, or wheelchair)  2 receives assistance in going to the toilet or in cleaning self or in arranging clothes afterwards or in use of night bedpan or commode  3 doesn’t use a toilet |  |  |
| E4 **Transfer** | 1 gets in and out of bed as well as in and out of a chair without assistance (may use object for support such as cane or walker)  2 gets in and out of bed or chair with assistance  3 bedridden |  |  |
| E5 **Continence** | 1 has complete control of urination and bowel movement without assistance  2 has occasional ‘accidents’  3 supervision helps keep urine or bowel control; catheter is used or elder is incontinent |  |  |
| E6 **Feeding** | 1 feeds self without assistance  2 feeds self, with some help  3 receives assistance in feeding or is fed partly or completely intravenously |  |  |

| F. PERSONAL BACKGROUND | |  | Code |
| --- | --- | --- | --- |
| F1 How many years did you attend school? | _______ |  |  |
| F2 What was your main occupation before age 60? | 0 professional and technical personnel  1 governmental, institutional or managerial personnel  2 agriculture, forest, animal husbandry  3 fishery worker  4 industrial worker  5 commercial or service worker  6 military personnel  7 housework  8 other, please specify:______________ |  |  |
| F3.1 What is your main means of financial support? | 1 retirement wages  2 spouse 3 children  4 grandchildren 5 other relative  6 from local government or community  7 work  8 other, please specify:______________ |  |  |

| F3.2 Could your main financial support maintain your daily cost? | | 1 yes 2 no 3 so so  4 don’t know | | | |  |  | | | |
| --- | --- | --- | --- | --- | --- | --- | --- | --- | --- | --- |
| F3.3 What is your other means of financial support? (multiple choices but limit to 5 choices) | | 1 retirement wages  2 spouse 3 children  4 grandchildren 5 other relative  6 from local government or community  7 work  8 other, please specify:______________ | | | |  |  | | | |
| F3.2 Could all of your financial support maintain your daily cost? | | 1 yes 2 no 3 so so  4 don’t know | | | |  |  | | | |
| F4.1 Current marital status: | | 1 married and living with spouse  2 separated  3 divorced  4 widowed  5 never married | | | |  |  | | | |
| F4.2 How many times have you been married? | | __________, *if answer 0-- jump to F5* | | | |  |  | | | |
| F4.3 Please tell me your marriage history | | your age at this marriage | status of this marriage | age at marriage dissolution | good relationship? |  | age at this marriage | status | age at marriage dissolution | relationship |
| *(‘age at marriage dissolution’ to be answered only by divorced or widowed people)* | 1^st^ marriage |  | 1 married  2 divorced  3 widowed |  | 1 good  2 so so  3 bad |  |  |  |  |  |
|  | 2^nd^ marriage |  | 1 married  2 divorced  3 widowed |  | 1 good  2 so so  3 bad |  |  |  |  |  |
|  | 3^rd^ marriage |  | 1 married  2 divorced  3 widowed |  | 1 good  2 so so  3 bad |  |  |  |  |  |
|  | 4^th^ marriage |  | 1 married  2 divorced  3 widowed |  | 1 good  2 so so  3 bad |  |  |  |  |  |
| F4.4 How many years did your last spouse attend school? | | __________ | | | |  |  | | | |
| F4.5 What was your last spouse’s main occupation before age 60? | | 0 professional and technical personnel  1 governmental, institutional or managerial personnel  2 agriculture, forest, animal husbandry  3 fishery worker  4 industrial worker  5 commercial or service worker  6 military personnel  7 housework  8 other, please specify:______________ | | | |  |  | | | |

| F5 When you are sick, who usually takes care of you? | 1 children and/or spouse  2 other family members  3 friends  4 live-in caregiver  5 social service  6 nobody |  |  |
| --- | --- | --- | --- |
| F6.1 Can you get adequate medical service when you are sick? | 1 yes 2 no 3 never been sick |  |  |
| F6.2 Could you get adequate medical service when you were sick at around age 60? | 1 yes 2 no 3 never was sick |  |  |
| F6.3 Could you get adequate medical service when you were sick in childhood? | 1 yes 2 no 3 never was sick |  |  |
| F6.4 Who mainly pay for your medical cost? | 1 public medical health 2 self  3 family and children 4 other |  |  |
| F6.5 What’s the main reason that you didn’t go to hospital when it was necessary? | 1 no money to pay for  2 far away 3 inconvenient in movement  4 nobody to go with 5 others |  |  |
| F6.6 Did you frequently go to bed hungry as a child? | 1 yes 2 no |  |  |
| F7.1 Is your mother alive? | 1 yes 2 no |  |  |
| F7.2 If so, how old is she?  If not, how old was she when she died? | _______  _______ |  |  |
| F7.3 If she is dead, how old were you when she died? | _______ |  |  |
| F8.1 Is your father alive? | 1 yes 2 no |  |  |
| F8.2 If so, how old is he?  If not, how old was he when he died? | _______    _______ |  |  |
| F8.3 If he is dead, how old were you when he died? | _______ |  |  |
| F8.4 The main occupation of your father before age 60 | 0 professional and technical personnel  1 governmental, institutional or managerial personnel  2 agriculture, forest, animal husbandry  3 fishery worker  4 industrial worker  5 commercial or service worker  6 military personnel  7 housework  8 other |  |  |

| F9 How many biological siblings, including those who have died, do you have? | | _______ | | | | | | | | | | | |  |  | | | | | | | |  | |
| --- | --- | --- | --- | --- | --- | --- | --- | --- | --- | --- | --- | --- | --- | --- | --- | --- | --- | --- | --- | --- | --- | --- | --- | --- |
| F9.1 What is your birth order among all of your biological siblings? | | _______ | | | | | | | | | | | |  |  | | | | | | | |  | |
| F9.2 Please tell me about your biological brothers and sisters who live elsewhere or have died, by birth order.  **Sex:** 1 male 2 female  **living or not:**  1 yes 2 no  **age:** *if alive, fill in the age at present. If dead, fill in the age of death.*  **frequent visits?**  1 yes 2 no  **residence:**  1 in the same village/neighborhood  2 in the same township/district  3 in the same county/city  4 in a county/city nearby  5 elsewhere  8 unknown  *(If alive, fill in the place where she/he lives at present. If dead, fill in the place where she/he lived before her/his death.)* | | birth order | name | | sex | | alive or not | | age | | frequent visits? | | residence |  | birth order | | sex | | alive or not | age | | visits? | residence |  |
|  |  |  |  | |  | |  | |  | |  | |  |  |  | |  | |  |  | |  |  |  |
|  |  |  |  | |  | |  | |  | |  | |  |  |  | |  | |  |  | |  |  |  |
|  |  |  |  | |  | |  | |  | |  | |  |  |  | |  | |  |  | |  |  |  |
|  |  |  |  | |  | |  | |  | |  | |  |  |  | |  | |  |  | |  |  |  |
|  |  |  |  | |  | |  | |  | |  | |  |  |  | |  | |  |  | |  |  |  |
|  |  |  |  | |  | |  | |  | |  | |  |  |  | |  | |  |  | |  |  |  |
|  |  |  |  | |  | |  | |  | |  | |  |  |  | |  | |  |  | |  |  |  |
|  |  |  |  | |  | |  | |  | |  | |  |  |  | |  | |  |  | |  |  |  |
|  |  |  |  | |  | |  | |  | |  | |  |  |  | |  | |  |  | |  |  |  |
|  |  |  |  | |  | |  | |  | |  | |  |  |  | |  | |  |  | |  |  |  |
| **name** | **address, if alive and aged 80 or over** |  |  | |  | |  | |  | |  | |  |  |  | |  | |  |  | |  |  |  |
|  |  |  |  | |  | |  | |  | |  | |  |  |  | |  | |  |  | |  |  |  |
|  |  |  |  | |  | |  | |  | |  | |  |  |  | |  | |  |  | |  |  |  |
|  |  |  |  | |  | |  | |  | |  | |  |  |  | |  | |  |  | |  |  |  |
|  |  |  |  | |  | |  | |  | |  | |  |  |  | |  | |  |  | |  |  |  |
|  |  |  |  | |  | |  | |  | |  | |  |  |  | |  | |  |  | |  |  |  |
| F10 How many children, including those who have died, do you have? | | _______ | | | | | | | | | | | |  |  | | | | | | | |  | |
| F10.1 Your age at first delivery | | ________ | | | | | | | | | | | |  |  | | | | | | | |  | |
| F10.2 Your age at last delivery | | ________ | | | | | | | | | | | |  |  | | | | | | | |  | |
| F10.3 Please tell me about your children who live elsewhere or have died, by birth order.  **sex:**  1 male 2 female  **alive or not:**  1 alive 2 dead 3 unknown  **age at present:**  *If alive, fill in the age at present. If dead, how old would she/he be today?*  **frequent visits?**  1 yes 2 no  **residence:**  1 in the same village/neighbourhood  2 in the same township/district  3 in the same county/city  4 in the county/city nearby  5 elsewhere  8 unknown  *(If alive, fill in the place where she/he lives at present. If dead, fill in the place where she/he lived before her/his death.)* | | name | | sex | | alive? | | age at present | | frequent visits? | | residence | |  | sex | alive? | | age at present | | | frequent visits? | | residence | |
|  |  |  | |  | |  | |  | |  | |  | |  |  |  | |  | | |  | |  | |
|  |  |  | |  | |  | |  | |  | |  | |  |  |  | |  | | |  | |  | |
|  |  |  | |  | |  | |  | |  | |  | |  |  |  | |  | | |  | |  | |
|  |  |  | |  | |  | |  | |  | |  | |  |  |  | |  | | |  | |  | |
|  |  |  | |  | |  | |  | |  | |  | |  |  |  | |  | | |  | |  | |
|  |  |  | |  | |  | |  | |  | |  | |  |  |  | |  | | |  | |  | |
|  |  |  | |  | |  | |  | |  | |  | |  |  |  | |  | | |  | |  | |
|  |  |  | |  | |  | |  | |  | |  | |  |  |  | |  | | |  | |  | |
|  |  |  | |  | |  | |  | |  | |  | |  |  |  | |  | | |  | |  | |
|  |  |  | |  | |  | |  | |  | |  | |  |  |  | |  | | |  | |  | |
|  |  |  | |  | |  | |  | |  | |  | |  |  |  | |  | | |  | |  | |
|  |  |  | |  | |  | |  | |  | |  | |  |  |  | |  | | |  | |  | |
|  |  |  | |  | |  | |  | |  | |  | |  |  |  | |  | | |  | |  | |
|  | |  | |  | |  | |  | |  | |  | |  |  |  | |  | | |  | |  | |

| **G. Objective examination and illnesses** | | | Code |
| --- | --- | --- | --- |
| G1 Can the interviewee see a break in the circle on the cardboard sheet when lit by a flashlight and distinguish where the break is located? | 1 can see and distinguish  2 cannot see 3 blind |  |  |
| G2.1 How many natural teeth does the interviewee have? | ______ |  |  |
| G2.2 Does the interviewee have false teeth? | 1 yes 2 no |  |  |
| G3 Hemisphere dominance: | 1 right-handed  2 left-handed |  |  |

| G4 Can the interviewee use chopsticks to eat | 1 yes 2 no |  |  |
| --- | --- | --- | --- |
| G5. Blood pressure  G5.1 Systolic  G5.2 Diastolic | ________mm mercury  ________mm mercury |  |  |
|
|
| G6 Rhythm of heart | 1 regular 2 irregular |  |  |
| G7 Heart rate | ________beats/min |  |  |
| G8 Upper extremities - can interviewee put  G8.1 Hand behind neck | 1 right 2 left  3 both 4 neither |  |  |
| G8.2 Hand behind lower back | 1 right 2 left  3 both 4 neither |  |  |
| G9 Can the interviewee stand up from a chair? | 1 yes, without using hands  2 yes, using hands  3 no |  |  |
| G10 Weight | ______ kg |  |  |
| G11 Was the interviewee able to pick up a book from the floor? | 1 yes, standing  2 yes, sitting 3 no |  |  |
| G12 Was the interviewee able to turn around 360° without help?  *If yes, please count the number of steps required to finish the turn.* | ______steps *(if no, fill in ‘88’)* |  |  |
| G13 How many times have you suffered from serious illness which required hospitalization or caused you to be bedridden at home in the past 2 years? | ______ *(if no illnesses, fill in ‘00’; if permanently bedridden, fill in ‘88’)* |  |  |
| G14 What kind of diseases suffered from ?(ref. code in G15) | First time: disease_____ days_____  Second time: disease_____ days_____  Last time: disease_____ days_____ |  | ,  ,  , |

| G15 Are you suffering from any of the following | yes or no  1 yes  2. no  3 don’t know | Diagnosed by hospital?  1 yes 2 no | disability in daily life  1 rather serious  2 more or less  3 no | With disease or not? | Diagnosed by hospital or not? | disability |
| --- | --- | --- | --- | --- | --- | --- |
| Hypertension |  |  |  |  |  |  |
| Diabetes |  |  |  |  |  |  |
| Heart diseases |  |  |  |  |  |  |
| Stroke , cerebrovascular disease |  |  |  |  |  |  |
| Bronchitis, emphysema, asthma, pneumonia |  |  |  |  |  |  |
| Pulmonary tuberculosis |  |  |  |  |  |  |
| Cataract |  |  |  |  |  |  |
| Glaucoma |  |  |  |  |  |  |
| Cancer |  |  |  |  |  |  |
| Prostate tumor |  |  |  |  |  |  |
| Gastric or duodenal ulcer |  |  |  |  |  |  |
| Parkinson’s disease |  |  |  |  |  |  |
| Bedsore |  |  |  |  |  |  |
| Arthritis |  |  |  |  |  |  |
| Dementia |  |  |  |  |  |  |
| Others, please specify: ___________ |  |  |  |  |  |  |

| H. QUESTIONS FOR INTERVIEWER | | | Code |
| --- | --- | --- | --- |
| H1 Was the interviewee able to hear what you said? | 1 yes, without hearing aid  2 yes, but needs hearing aid  3 partly, despite hearing aid  4 no |  |  |
| H2.1 Did interviewee able to participate physical check during interview? | 1 yes 2 no 3 partially able to |  |  |
| H2.2 If no or partially able, please give reason: | 1 visually impaired, but can hear  2 hearing impaired, but can see  3 visually and hearing impaired  4 paralyzed  5 did not wish to participate  6 could not understand because of cognitive impairment  7 not able to participate at the moment because of some temporary illness such as a cold  8 other (please explain):  ________________________ |  |  |
| H3 The interviewee was: | 1 surprisingly healthy (almost no obvious ailments)  2 relatively healthy (only minor ailments)  3 moderately ill (moderate degrees of major ailments or illnesses)  4 very ill (major ailments or diseases, bedridden, etc.) |  |  |
| H4 Date of birth printed on the household booklet | ______year ____month ____day |  |  |
| H4.1 Was the date of birth printed on the household booklet the same as the self-reported age? | 1 no 2 yes 3 no self-reporting |  |  |
| H4.2 If not, which one do you consider correct? | 1 self-reported age  2 household booklet  3 not sure |  |  |
| H5 Please write down the evidence for confirming the interviewee’s age-reporting:  *(Regardless of whether or not there is self-reporting, you should confirm the age of the interviewee. If there is not enough space to write down your confirmation, please use the last page of this questionnaire)*. | __________________________ |  |  |
| H6 Have you checked whether you have failed to ask a question？ | 1 yes 2 no |  |  |
| H7 Did anyone help the interviewee to answer any question？ | 1 yes 2 no |  |  |
| H7.1 If yes, please check whether you have marked ‘x’ in the  of the third column for those questions answered by people other than the interviewee. Please indicate who mainly helped to answer those questions. | 1 spouse  2 child or spouse of child  3 grandchild or spouse of grandchild  4 great grandchild or spouse of  great grandchild  5 sibling  6 parent or parent-in-law 7 live-in caregiver  8 other |  |  |
| H8 Interviewee’s personal photo | 1 yes 2 no |  |  |

| Special observations | | | |  |
| --- | --- | --- | --- | --- |
|  | | | |  |
|  | **I. Special questions (only applicable to those aged 105 or above)** | | Code | |
|  | ***Note to all persons who help to answer the questions listed below:***  ***According to article 14 of chapter 3 of the Law on Statistics, all information collected in this survey will be treated as strictly confidential. We will not tell anyone, including the elder him/herself, that you have helped to provide us information by answering the following questions, and your name will not be written down anywhere. The information collected here is purely for scientific research and nobody except qualified researchers will have access to this information. There will be NO connection between information collected here and the personal honour of the elder or any benefits she/he receives.***  ***The elder's name: Sex: Code: Self-reported age:*** | | | |
|  | S1 Information obtained from the elder’s neighbors:  What is your opinion about this?  *(Present these possibilities and ask the respondent to chose one. The respondent should freely chose one based on the facts he or she knows.)* | 1 I do not think the elder’s age is correct.  2 I have doubts about the elder’s age.  3 Perhaps the elder’s age is correct, perhaps not – I do not know.  4 I suppose the elder’s age is correct, but I do not know for sure.  5 I am absolutely sure the elder’s age is correct.  *Whatever answer the respondent chooses please ask him or her to explain why:*  __________________________________ |  | |
|  | S2 Information obtained from the village leader or the neighborhood committee leader:  What is your opinion about this?  *(Present these possibilities and ask the respondent to chose one. The respondent should freely chose one based on the facts he or she knows.)* | 1 I do not think the elder’s age is correct.  2 I have doubts about the elder’s age.  3 Perhaps the elder’s age is correct, perhaps not – I do not know.  4 I suppose the elder’s age is correct, but I do not know for sure.  5 I am absolutely sure the elder’s age is correct.  *Whatever answer the respondent chooses please ask him or her to explain why:*  __________________________________ |  | |
|  | S3 Information obtained from the Aging Association officer:  What is your opinion about this?  *(Present these possibilities and ask the respondent to chose one. The respondent should freely chose one based on the facts he or she knows.)* | 1 I do not think the elder’s age is correct.  2 I have doubts about the elder’s age.  3 Perhaps the elder’s age is correct, perhaps not – I do not know.  4 I suppose the elder’s age is correct, but I do not know for sure.  5 I am absolutely sure the elder’s age is correct.  *Whatever answer the respondent chooses please ask him or her to explain why:*  __________________________________ |  | |
|  | S4 If there are genealogical records for the elder, please locate them and answer the following questions:  S4.1 Birth date of the elder:  S4.2 Date of first marriage of the elder: | year ; month ; day ;  year ; month ; day ; | ,  ,  ,  , | |
