# Supplementary material for: Association of APOE ε4 genotype and lifestyle with cognitive function among Chinese adults aged 80 years and older: A cross-sectional study
Source: PLoS Med. 2021 Jun 1;18(6):e1003597. doi: 10.1371/journal.pmed.1003597 (PMC8168868; doi:10.1371/journal.pmed.1003597)
Supplement: S1 Fig — Adjustment: age at baseline, sex, residency, education level, APOE genotype, lifestyle factors (smoking, alcohol consumption, dietary pattern, and physical activity), activity of daily living, and 7 kinds of self-reported disease (COPD, tuberculosis, all-cause cancer, diabetes, hypertension, stroke, and cardiovascular disease). The logistics regression models with penalized splines evaluated nonlinear associations of cognitive impairment with weight and BMI; 2 cutoffs were identified (weight: less than 38 kg or higher than 50 kg, BMI: lower than 18 kg/m2 or higher than 21 kg/m2) above and below, in which there was no significant increase in the multitude of OR for cognitive impairment. APOE, apolipoprotein E; BMI, body mass index; COPD, chronic obstructive pulmonary disease; OR, odds ratio. (DOCX) [file pmed.1003597.s005.docx]

**S1 Fig The adjusted odds ratio of cognitive impairment of body weight and BMI in logistics regression models with penalized splines.**


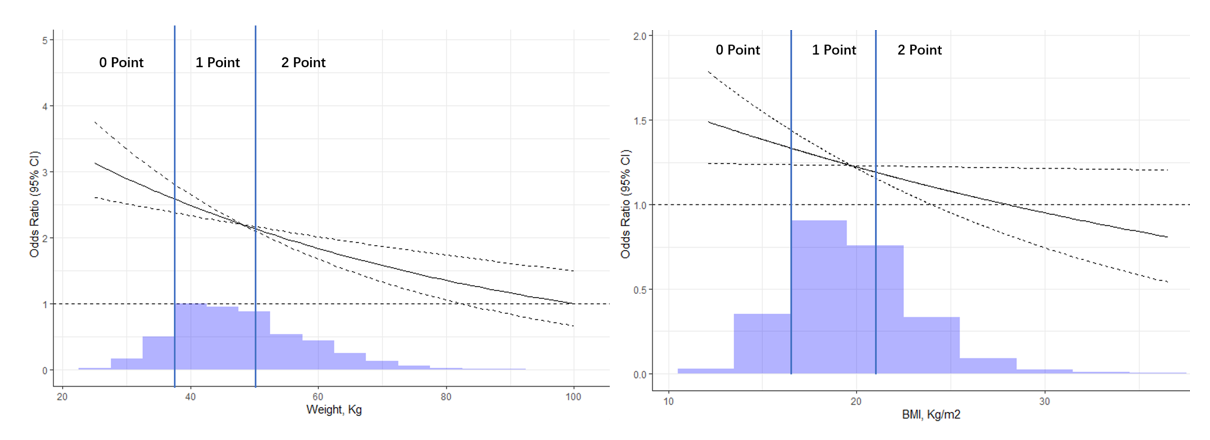


Adjustment: age at baseline, sex, residency, education level, *APOE* genotype, lifestyle factors (smoking, alcohol consumption, dietary pattern, physical activity), activity of daily living and seven kinds of self-reported disease (chronic obstructive pulmonary disease (COPD), tuberculosis, all-cause cancer, diabetes, hypertension, stroke and cardiovascular disease).

The logistics regression models with penalized splines evaluated non-linear associations of cognitive impairment with weight and BMI; two cut-offs were identified (weight: less than 38 kg or higher than 50 kg, BMI: lower than 18 kg/m^2^ or higher than 21 kg/m^2^) above and below which there was no significant increase in the multitude of odd ratio for cognitive impairment.
