# Supplementary material for: Association of APOE ε4 genotype and lifestyle with cognitive function among Chinese adults aged 80 years and older: A cross-sectional study
Source: PLoS Med. 2021 Jun 1;18(6):e1003597. doi: 10.1371/journal.pmed.1003597 (PMC8168868; doi:10.1371/journal.pmed.1003597)
Supplement: S2 Fig — Adjustment: age at baseline, sex, residency, education level, APOE genotype, activity of daily living, and 7 kinds of self-reported disease (COPD, tuberculosis, all-cause cancer, diabetes, hypertension, stroke, and cardiovascular disease). The logistics regression models with penalized splines evaluated nonlinear associations of cognitive impairment with lifestyle score and modifiable factor score; 2 cutoffs were identified (lifestyle score: less than 6 or higher than 7; modifiable factor score: less than 5 or higher than 8) above and below, in which there was no significant increase in the multitude of OR for cognitive impairment. APOE, apolipoprotein E; COPD, chronic obstructive pulmonary disease; OR, odds ratio. (DOCX) [file pmed.1003597.s006.docx]

**S2 Fig The adjusted odds ratio of cognitive impairment for lifestyle score and modifiable factor score in logistics regression models with penalized splines.**


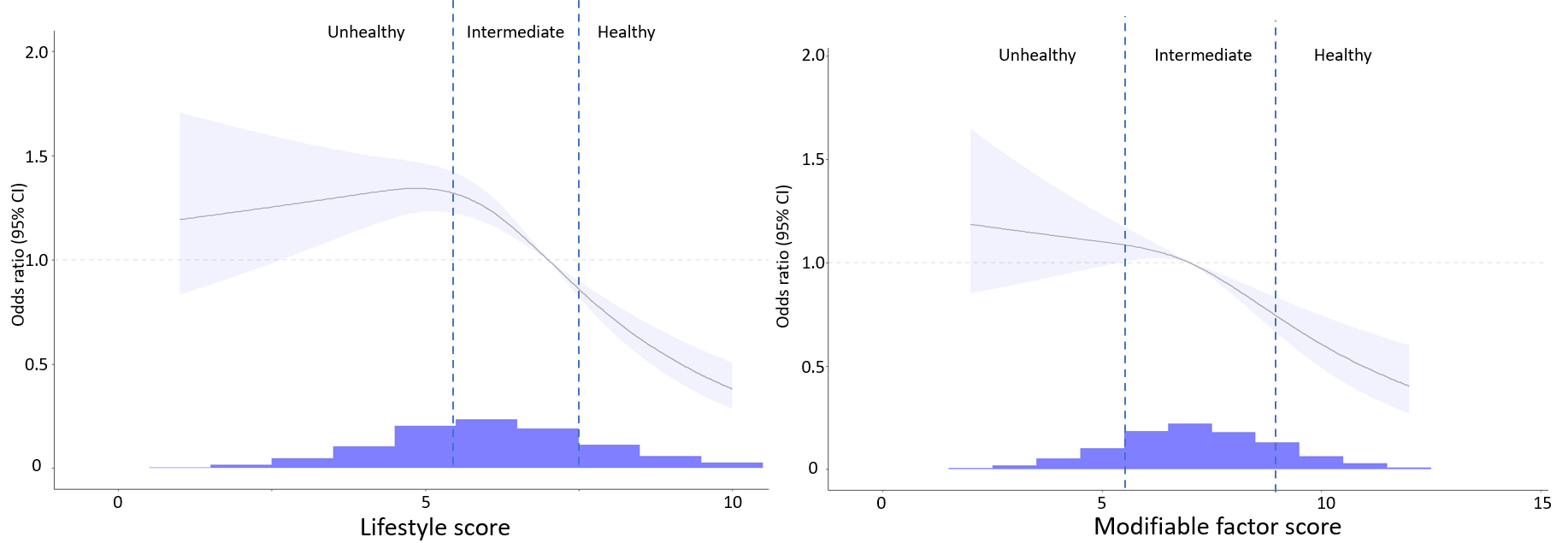


Adjustment: age at baseline, sex, residency, education level, *APOE* genotype, activity of daily living and seven kinds of self-reported disease (chronic obstructive pulmonary disease (COPD), tuberculosis, all-cause cancer, diabetes, hypertension, stroke and cardiovascular disease).

The logistics regression models with penalized splines evaluated non-linear associations of cognitive impairment with lifestyle score and modifiable factor score; two cut-offs were identified (lifestyle score: less than 6 or higher than 7; Modifiable factor score: less than 5 or higher than 8) above and below which there was no significant increase in the multitude of odd ratio for cognitive impairment.
