# Supplementary material for: Association of APOE ε4 genotype and lifestyle with cognitive function among Chinese adults aged 80 years and older: A cross-sectional study
Source: PLoS Med. 2021 Jun 1;18(6):e1003597. doi: 10.1371/journal.pmed.1003597 (PMC8168868; doi:10.1371/journal.pmed.1003597)
Supplement: S1 Table — Cognitive impairment: Cognitive impairment was defined by MMSE scores less than 18. Adjustment: age at baseline, sex, residency, education level, APOE genotype, activity of daily living, and 7 kinds of self-reported disease (COPD, tuberculosis, all-cause cancer, diabetes, hypertension, stroke, and cardiovascular disease). APOE, apolipoprotein E; COPD, chronic obstructive pulmonary disease; MMSE, Mini-Mental State Examination. (DOCX) [file pmed.1003597.s007.docx]

**S1 Table Association between single lifestyle factors and cognitive impairment stratified by *APOE* genotype**

| **Independent variable** | **Adjusted OR of cognitive impairment, (95% CI)** | | ***P* for Interaction** |
| --- | --- | --- | --- |
|  | **APOE ε4 carriers** | ***APOE* ε4 non-carriers** |  |
| **Smoking** |  |  |  |
| Current | *Reference* | |  |
| Former | 0.97 (0.70, 1.34) | 0.94 (0.48, 1.84) | 0.80 |
| Never | 1.10 (0.84, 1.43) | 0.96 (0.57, 1.64) | 0.83 |
| **Drinking** |  |  |  |
| Heavy | *Reference* | |  |
| Moderate | 0.74 (0.50, 1.11) | 1.31 (0.50, 3.41) | 0.44 |
| Never | 0.98 (0.75, 1.27) | 1.59 (0.85, 2.97) | 0.36 |
| **Physical activity** |  |  |  |
| Never | *Reference* | |  |
| Former | 0.93 (0.69, 1.24) | 0.58 (0.30, 1.13) | 0.27 |
| Current | 0.69 (0.56, 0.84) | 0.49 (0.32, 0.77) | 0.19 |
| **Dietary pattern** |  |  |  |
| Unfavorable | *Reference* |  |  |
| Intermediate | 0.68 (0.60, 0.85) | 0.44 (0.30, 0.67) | 0.078 |
| Favorable | 0.41 (0.69, 0.54) | 0.36 (0.22, 0.58) | 0.52 |
| **Body weight** |  |  |  |
| <38 | *Reference* | |  |
| 38-50 | 1.01 (0.82, 1.24) | 0.80 (0.35, 1.28) | 0.37 |
| >50 | 0.88 (0.69, 1.13) | 0.99 (0.56, 1.75) | 0.77 |

Cognitive impairment: cognitive impairment was defined by Mini-Mental State Examination scores less than 18.

Model adjusted for age, sex, residence, education level, marital status, *APOE* genotype, lifestyle factors (smoking, alcohol consumption, physical activity, body weight and dietary pattern), activity of daily living, and seven kinds of self-reported disease (chronic obstructive pulmonary disease, tuberculosis, cancer, diabetes, hypertension, stroke and cardiovascular disease).
