# Supplementary material for: Association of APOE ε4 genotype and lifestyle with cognitive function among Chinese adults aged 80 years and older: A cross-sectional study
Source: PLoS Med. 2021 Jun 1;18(6):e1003597. doi: 10.1371/journal.pmed.1003597 (PMC8168868; doi:10.1371/journal.pmed.1003597)
Supplement: S2 Table — Model was adjusted for age at baseline, baseline MMSE score, sex, residency, education level, marital status, APOE genotype, lifestyle profile, activity of daily living, and 7 kinds of self-reported disease (COPD, tuberculosis, all-cause cancer, diabetes, hypertension, stroke, and cardiovascular disease). APOE, apolipoprotein E; COPD, chronic obstructive pulmonary disease; MMSE, Mini-Mental State Examination. (DOCX) [file pmed.1003597.s008.docx]

**S2 Table Associations of cognitive decline with *APOE* ε4 genotype and lifestyle profiles (N=3136 with 1564 decline)**

|  | **Logistic regression**  **OR of cognitive decline, (95% CI)** | | | |
| --- | --- | --- | --- | --- |
|  | **Partially adjusted model*** | ***P* value** | **Adjusted model**** | ***P* value** |
| ***APOE* ε4 genotype** |  |  |  |  |
| ε4 carriers | *Reference* |  | *Reference* |  |
| Non**-**carriers | 0.87 (0.72, 1.05) | 0.14 | 0.72 (0.54, 0.95) | 0.033 |
| **Lifestyle profile** |  |  |  |  |
| Unhealthy | *Reference* |  | *Reference* |  |
| Intermediate | 0.73 (0.58, 0.91) | 0.007 | 0.75 (0.60, 0.94) | 0.027 |
| **Lifestyle Profile by APOE genotype** | |  |  |  |
| *APOE* ε4 carriers |  |  |  |  |
| Unhealthy lifestyle | *Reference* |  | *Reference* |  |
| Healthy lifestyle | 0.78 (0.67, 0.93) | 0.005 | 0.77 (0.60, 0.98) | 0.035 |
| *APOE* ε4 Non-carriers |  |  |  |  |
| Unhealthy lifestyle | *Reference* |  | *Reference* |  |
| Healthy lifestyle | 0.76 (0.49, 1.25) | 0.41 | 0.74 (0.46, 1.22) | 0.31 |

*****Model was adjusted for baseline MMSE score.

*****Model was adjusted for age at baseline, baseline MMSE score, sex, residency, education level, marital status, *APOE* genotype, lifestyle profile, activity of daily living and seven kinds of self-reported disease (chronic obstructive pulmonary disease (COPD), tuberculosis, all-cause cancer, diabetes, hypertension, stroke and cardiovascular disease).
