# Supplementary material for: Association of APOE ε4 genotype and lifestyle with cognitive function among Chinese adults aged 80 years and older: A cross-sectional study
Source: PLoS Med. 2021 Jun 1;18(6):e1003597. doi: 10.1371/journal.pmed.1003597 (PMC8168868; doi:10.1371/journal.pmed.1003597)
Supplement: S4 Table — Model was adjusted for age at baseline, sex, residency, education level, marital status, APOE genotype, lifestyle profile, activity of daily living, and 7 kinds of self-reported disease (COPD, tuberculosis, all-cause cancer, diabetes, hypertension, stroke, and cardiovascular disease). APOE, apolipoprotein E; COPD, chronic obstructive pulmonary disease. (DOCX) [file pmed.1003597.s010.docx]

**S4 Table Sensitivity analysis associations of cognitive function with *APOE* ε4 genotype and lifestyle profiles: excluding the participants with deafness or blindness (N=6,038)**

|  | **Logistic regression, OR of cognitive impairment (95% CI)** | | | |
| --- | --- | --- | --- | --- |
|  | **Unadjusted model** | ***P* value** | **Adjusted model*** | ***P* value** |
| ***APOE* ε4 genotype** |  |  |  |  |
| ε4 carriers | *Reference* |  | *Reference* |  |
| Non**-**carriers | 0.84 (0.71, 0.99) | 0.049 | 0 .83 (0.69, 0.98) | 0.007 |
| **Lifestyle profile** |  |  |  |  |
| Unhealthy | *Reference* |  | *Reference* |  |
| Intermediate | 0.75 (0.65, 0.86) | <0.001 | 0.73 (0.63, 0.85) | <0.001 |
| Healthy | 0.41 (0.33, 0.50) | <0.001 | 0.45 (0.36, 0.56) | <0.001 |

*Model was adjusted for age at baseline, sex, residency, education level, marital status, *APOE* genotype, lifestyle profile, activity of daily living and seven kinds of self-reported disease (chronic obstructive pulmonary disease (COPD), tuberculosis, all-cause cancer, diabetes, hypertension, stroke and cardiovascular disease).
