# Supplementary material for: Association of APOE ε4 genotype and lifestyle with cognitive function among Chinese adults aged 80 years and older: A cross-sectional study
Source: PLoS Med. 2021 Jun 1;18(6):e1003597. doi: 10.1371/journal.pmed.1003597 (PMC8168868; doi:10.1371/journal.pmed.1003597)
Supplement: S5 Table — Model was adjusted for age at baseline, sex, residency, education level, marital status, APOE genotype, lifestyle profile, activity of daily living, and 7 kinds of self-reported disease (COPD, tuberculosis, all-cause cancer, diabetes, hypertension, stroke and cardiovascular disease). APOE, apolipoprotein E; COPD, chronic obstructive pulmonary disease; MMSE, Mini-Mental State Examination. (DOCX) [file pmed.1003597.s011.docx]

**S5 Table Sensitivity analysis: associations of cognitive function with *APOE* ε4 genotype and lifestyle profiles: excluding the participants with MMSE score equal to zero (N=5,655)**

|  | **Logistic regression, OR of cognitive impairment (95% CI)** | | | |
| --- | --- | --- | --- | --- |
|  | **Unadjusted model** | ***P* value** | **Adjusted model*** | ***P* value** |
| ***APOE* ε4 genotype** |  |  |  |  |
| ε4 carriers | *Reference* |  | *Reference* |  |
| Non**-**carriers | 0.78 (0.65, 0.94) | 0.009 | 0.69 (0.39, 0.93) | 0.007 |
| **Lifestyle profile** |  |  |  |  |
| Unhealthy | *Reference* |  | *Reference* |  |
| Intermediate | 0.76 (0.66, 0.89) | <0.001 | 0.72 (0.60, 0.85) | <0.001 |
| Healthy | 0.39 (0.31, 0.48) | <0.001 | 0.49 (0.38, 0.63) | <0.001 |

*Model was adjusted for age at baseline, sex, residency, education level, marital status, *APOE* genotype, lifestyle profile, activity of daily living and seven kinds of self-reported disease (chronic obstructive pulmonary disease (COPD), tuberculosis, all-cause cancer, diabetes, hypertension, stroke and cardiovascular disease).
