# Supplementary material for: Association of APOE ε4 genotype and lifestyle with cognitive function among Chinese adults aged 80 years and older: A cross-sectional study
Source: PLoS Med. 2021 Jun 1;18(6):e1003597. doi: 10.1371/journal.pmed.1003597 (PMC8168868; doi:10.1371/journal.pmed.1003597)
Supplement: S7 Table — Model was adjusted for age at baseline, sex, residency, education level, marital status, APOE genotype, lifestyle profile, activity of daily living, and 7 kinds of self-reported disease (COPD, tuberculosis, all-cause cancer, diabetes, hypertension, stroke, and cardiovascular disease). APOE, apolipoprotein E; COPD, chronic obstructive pulmonary disease; MMSE, Mini-Mental State Examination. (DOCX) [file pmed.1003597.s013.docx]

**S7 Table Sensitivity analysis: associations of cognitive function with *APOE* ε4 genotype and lifestyle profiles:** **defining cognitive impairment with different MMSE cutoff and using the education-adjusted outcome**

|  | **Logistic regression, OR of cognitive impairment, (95% CI)** | | | | | | | |
| --- | --- | --- | --- | --- | --- | --- | --- | --- |
| ***Definition of Cognitive impairment*** | <16 | *P* value | <21 | *P* value | <25 | *P* value | Education-adjusted | *P* value |
| ***Unadjusted model*** |  |  |  |  |  |  |  |  |
| ***APOE* ε4 genotype** |  |  |  |  |  |  |  |  |
| ε4 carriers | *Reference* |  | *Reference* |  | *Reference* |  | *Reference* |  |
| Non**-**carriers | 0.77 (0.63, 0.95) | 0.013 | 0.82 (0.69, 0.95) | 0.013 | 0.88 (0.76, 1.04) | 0.15 | 0.85 (0.72, 1.00) | 0.062 |
| **Lifestyle profile** |  |  |  |  |  |  |  |  |
| Unhealthy | *Reference* |  | *Reference* |  | *Reference* |  | *Reference* |  |
| Intermediate | 0.71 (0.60, 0.84) | <0.001 | 0.72 (0.63, 0.83) | <0.001 | 0.70 (0.61, 0.80) | <0.001 | 0.71 (0.62, 0.83) | <0.001 |
| Healthy | 0.43 (0.33, 0.56) | <0.001 | 0.41 (0.34, 0.50) | <0.001 | 0.42 (0.35, 0.50) | <0.001 | 0.44 (0.35, 0.55) | <0.001 |
| ***Adjusted model**** |  |  |  |  |  |  |  |  |
| ***APOE* ε4 genotype** |  |  |  |  |  |  |  |  |
| ε4 carriers | *Reference* |  | *Reference* |  | *Reference* |  | *Reference* |  |
| Non**-**carriers | 0.69 (0.39 to 0.93) |  | 0.80 (0.67, 0.96) |  | 0.87 (0.74, 1.03) |  | 0.83 (0.69, 0.99) | 0.046 |
| **Lifestyle profile** |  |  |  |  |  |  |  |  |
| Unhealthy | *Reference* |  | *Reference* |  | *Reference* |  | *Reference* |  |
| Intermediate | 0.72 (0.60, 0.85) |  | 0.68 (0.59, 0.79) |  | 0.65 (0.57, 0.75) |  | 0.71 (0.62, 0.83) | <0.001 |
| Healthy | 0.49 (0.38, 0.63) |  | 0.42 (0.34, 0.52) |  | 0.42 (0.34, 0.51) |  | 0.44 (0.35, 0.55) | <0.001 |

*Model was adjusted for age at baseline, sex, residency, education level, marital status, *APOE* genotype, lifestyle profile, activity of daily living and seven kinds of self-reported disease (chronic obstructive pulmonary disease (COPD), tuberculosis, all-cause cancer, diabetes, hypertension, stroke and cardiovascular disease).
