# Supplementary material for: Association of APOE ε4 genotype and lifestyle with cognitive function among Chinese adults aged 80 years and older: A cross-sectional study
Source: PLoS Med. 2021 Jun 1;18(6):e1003597. doi: 10.1371/journal.pmed.1003597 (PMC8168868; doi:10.1371/journal.pmed.1003597)
Supplement: S8 Table — Model was adjusted for age at baseline, sex, residency, education level, marital status, APOE genotype, lifestyle profile, activity of daily living, and 7 kinds of self-reported disease (COPD, tuberculosis, all-cause cancer, diabetes, hypertension, stroke, and cardiovascular disease). APOE, apolipoprotein E; COPD, chronic obstructive pulmonary disease. (DOCX) [file pmed.1003597.s014.docx]

**S8 Table Sensitivity analysis:** **associations of cognitive function with *APOE* ε4 genotype and lifestyle profiles: adding the blood pressure and diabetes in the lifestyle score**

|  | **Logistic regression** | | | |
| --- | --- | --- | --- | --- |
|  | **Unadjusted Odds Ratio (95% CI)** | ***P* value** | **Adjusted Odds Ratio* (95% CI)** | ***P* value** |
| **Modifiable factors** |  |  |  |  |
| Unhealthy | *Reference* | | | |
| Intermediate | 0.82 (0.71, 0.95) | 0.006 | 0.81 (0.70, 0.95) | 0.008 |
| Healthy | 0.52 (0.43, 0.62) | <0.001 | 0.56 (0.45, 0.68) | <0.001 |
| ***Modifiable factors by APOE genotype*** | | |  |  |
| *APOE* ε4 carriers |  |  |  |  |
| Unhealthy | *Reference* | | | |
| Intermediate | 0.77 (0.56, 1.08) | 0.14 | 0.69 (0.48, 1.00) | 0.56 |
| Healthy | 0.43 (0.28, 0.68) | <0.001 | 0.39 (0.23, 0.64) | <0.001 |
| *APOE* ε4 non-carriers |  |  |  |  |
| Unhealthy | *Reference* | | | |
| Intermediate | 0.83 (0.71, 0.97) | 0.020 | 0.84 (0.71, 0.99) | 0.044 |
| Healthy | 0.53 (0.44, 0.65) | <0.001 | 0.59 (0.47, 0.74) | <0.001 |

*Model was adjusted for age at baseline, sex, residency, education level, marital status, *APOE* genotype, lifestyle profile, activity of daily living and seven kinds of self-reported disease (chronic obstructive pulmonary disease (COPD), tuberculosis, all-cause cancer, diabetes, hypertension, stroke and cardiovascular disease).
