# Supplementary material for: Association of APOE ε4 genotype and lifestyle with cognitive function among Chinese adults aged 80 years and older: A cross-sectional study
Source: PLoS Med. 2021 Jun 1;18(6):e1003597. doi: 10.1371/journal.pmed.1003597 (PMC8168868; doi:10.1371/journal.pmed.1003597)
Supplement: S9 Table — Model was adjusted for age at baseline, sex, residency, education level, marital status, APOE genotype, lifestyle profile, activity of daily living, and 7 kinds of self-reported disease (COPD, tuberculosis, all-cause cancer, diabetes, hypertension, stroke, and cardiovascular disease). APOE, apolipoprotein E; CDR, Clinical Dementia Rating; COPD, chronic obstructive pulmonary disease; MMSE, Mini-Mental State Examination. (DOCX) [file pmed.1003597.s015.docx]

**S9 Table Sensitivity analysis: associations of cognitive function with *APOE* ε4 genotype and lifestyle profiles:** **using ordinal logistics model and mapping the MMSE score to Clinical Dementia Rating as outcome/ using linear regression with MMSE score as outcome**

|  | **Ordinal logistic regression, OR of cognitive impairment (95% CI)** | | | | | **Linear regression, Coefficient of MMSE score, (95% CI)** | | | |
| --- | --- | --- | --- | --- | --- | --- | --- | --- | --- |
|  | **Unadjusted model** | | ***P* value** | **Adjusted* model** | ***P* value** | **Unadjusted model** | **P value** | **Adjusted model** | **P value** |
| ***APOE* ε4 genotype** |  | |  |  |  |  |  |  |  |
| ε4 carriers | *Reference* | |  | *Reference* |  | *Reference* |  | *Reference* |  |
| Non**-**carriers | 0.89 (0.78, 1.03) | | 0.12 | 0.87 (0.75, 1.00) | 0.050 | 0.44 (0.02, 0.87) | 0.041 | 0.46 (0.05, 0.88) | 0.026 |
| **Lifestyle profile** |  | |  |  |  |  |  |  |  |
| Unhealthy | *Reference* | |  | *Reference* |  | *Reference* |  | *Reference* |  |
| Intermediate | 0.76 (0.67, 0.85) | | <0.001 | 0.79 (0.70, 0.89) | <0.001 | 1.10 (0.74, 1.47) | <0.001 | 1.11 (0.76, 1.47) | <0.001 |
| Healthy | 0.47 (0.40, 0.55) | | <0.001 | 0.60 (0.50, 0.71) | <0.001 | 2.53 (2.09, 2.98) | <0.001 | 2.05 (1.60, 2.50) | <0.001 |
|  | | ***Lifestyle Profile*** | | |  |  |  |  |  |
| ***APOE* ε4 carriers** |  | |  |  |  |  |  |  |  |
| Unhealthy | *Reference* | |  | *Reference* |  | *Reference* |  | *Reference* |  |
| Intermediate | 0.82 (0.63, 1.09) | | 0.17 | 0.77 (0.58, 1.03) | 0.091 | 0.83 (-0.66, 1.72) | 0.070 | 1.01 (0.12, 1.89) | 0.025 |
| Healthy | 0.40 (0.27, 0.61) | | <0.001 | 0.45 (0.29, 0.70) | <0.001 | 3.11 (2.00, 4.22) | <0.001 | 2.66 (1.54, 3.79) | <0.001 |
| ***APOE* ε4 non-carriers** |  | |  |  |  |  |  |  |  |
| Unhealthy | *Reference* | |  | *Reference* |  | *Reference* |  | *Reference* |  |
| Intermediate | 0.74 (0.65, 0.84) | | <0.001 | 0.78 (0.69, 0.90) | <0.001 | 1.17 (0.77, 1.57) | <0.001 | 1.15 (0.76, 1.53) | <0.001 |
| Healthy | 0.47 (0.40, 0.57) | | <0.001 | 0.62 (0.52, 0.75) | <0.001 | 2.42 (1.93, 2.90) | <0.001 | 1.93 (1.43, 2.42) | <0.001 |

*Model was adjusted for age at baseline, sex, residency, education level, marital status, *APOE* genotype, lifestyle profile, activity of daily living and seven kinds of self-reported disease (chronic obstructive pulmonary disease (COPD), tuberculosis, all-cause cancer, diabetes, hypertension, stroke and cardiovascular disease).
