# Supplementary material for: Association of APOE ε4 genotype and lifestyle with cognitive function among Chinese adults aged 80 years and older: A cross-sectional study
Source: PLoS Med. 2021 Jun 1;18(6):e1003597. doi: 10.1371/journal.pmed.1003597 (PMC8168868; doi:10.1371/journal.pmed.1003597)
Supplement: S10 Table — Model was adjusted for age at baseline, sex, residency, education level, marital status, APOE genotype, lifestyle profile, activity of daily living, and 7 kinds of self-reported disease (COPD, tuberculosis, all-cause cancer, diabetes, hypertension, stroke, and cardiovascular disease). APOE, apolipoprotein E; COPD, chronic obstructive pulmonary disease. (DOCX) [file pmed.1003597.s016.docx]

**S10 Table Sensitivity analysis: associations of cognitive function with *APOE* ε4 genotype and lifestyle profiles: using Passion regression**

|  | **Poisson regression,**  **IRR of cognitive impairment, (95% CI)** | | | |
| --- | --- | --- | --- | --- |
|  | **Unadjusted model** | ***P* value** | **Adjusted* model** | ***P* value** |
| ***APOE* ε4 genotype** |  |  |  |  |
| ε4 carriers | *Reference* |  | *Reference* |  |
| Non**-**carriers | 0.89 (0.80, 0.99) | 0.033 | 0.89 (0.80, 0.99) | 0.035 |
| **Lifestyle profile** |  |  |  |  |
| Unhealthy | *Reference* |  | *Reference* |  |
| Intermediate | 0.83 (0.76, 0.90) | <0.001 | 0.82 (0.76, 0.90) | <0.001 |
| Healthy | 0.54 (0.47, 0.62) | <0.001 | 0.57 (0.49, 0.66) | <0.001 |
| ***Lifestyle profile*** |  |  |  |  |
| ***APOE* ε4** carriers |  |  |  |  |
| Unhealthy | *Reference* |  | *Reference* |  |
| Intermediate | 0.82 (0.68, 1.00) | 0.052 | 0.80 (0.66, 0.97) | 0.030 |
| Healthy | 0.46 (0.32, 0.68) | <0.001 | 0.48 (0.32, 0.72) | <0.001 |
| ***APOE* ε4 non-**carriers |  |  |  |  |
| Unhealthy | *Reference* |  | *Reference* |  |
| Intermediate | 0.83 (0.75, 0.91) | <0.001 | 0.82 (0.75, 0.91) | <0.001 |
| Healthy | 0.56 (0.48, 0.65) | <0.001 | 0.59 (0.50, 0.69) | <0.001 |

*Model was adjusted for age at baseline, sex, residency, education level, marital status, *APOE* genotype, lifestyle profile, activity of daily living and seven kinds of self-reported disease (chronic obstructive pulmonary disease (COPD), tuberculosis, all-cause cancer, diabetes, hypertension, stroke and cardiovascular disease).
